# Antibacterial Immunonegative Coating with Biocompatible Materials on a Nanostructured Titanium Plate for Orthopedic Bone Fracture Surgery

**DOI:** 10.34133/bmr.0070

**Published:** 2024-09-11

**Authors:** Jeong-Won Lee, Jung-Ah Cho, Yoo Jin Roh, Min Ae Han, Je-Un Jeong, Sivakumar Allur Subramanian, Eunho Kang, Jiwoo Yeom, Chang-Hun Lee, Sung Jae Kim

**Affiliations:** ^1^ Department of Mechanical Engineering, Chosun University, Gwangju 61452, Republic of Korea.; ^2^ Department of Orthopedic Surgery, Dongtan Sacred Hospital, Hallym University, Hwaseong, Republic of Korea.; ^3^ College of Transdisciplinary Studies, School of Undergraduate Studies, Daegu Gyeongbuk Institute of Science and Technology, Daegu 42988, Republic of Korea.; ^4^ Department of New Biology, Daegu Gyeongbuk Institute of Science and Technology, Daegu 42988, Republic of Korea.; ^5^ New Biology Research Center, Daegu Gyeongbuk Institute of Science and Technology, Daegu 42988, Republic of Korea.

## Abstract

Periprosthetic infections resulting from bacterial biofilm formation following surgical bone fracture fixation present important clinical challenges. Conventional orthopedic implant materials, such as titanium, are prone to biofilm formation. This study introduces a novel surface for orthopedic titanium plates, optimized for clinical application in human bone fractures. Leveraging nanostructure-based surface coating technology, the plate achieves an antibacterial/immunonegative surface using biocompatible materials, including poloxamer 407, epigallocatechin gallate, and octanoic acid. These materials demonstrate high biocompatibility and thermal stability after autoclaving. The developed plate, named antibacterial immunonegative surface, releases antibacterial agents and prevents adhesion between human tissue and metal surfaces. Antibacterial immunonegative surface plates exhibit low cell toxicity, robust antibacterial effects against pathogens such as *Staphylococcus aureus* and *Pseudomonas aeruginosa*, high resistance to biofilm formation on the implant surface and surrounding tissues, and minimal immune reaction in a rabbit femoral fracture model. This innovation holds promise for addressing periprosthetic infections and improving the performance of orthopedic implants.

## Introduction

Metal plates made of titanium alloy (TiAl6V4; hereafter referred to as Ti) are the most widely used medical implants for surgically treating bone fractures in current orthopedic medicine. The displaced bone fracture is properly reduced, followed by the use of a Ti plate for fixation with several screws across the fracture site. Implanted Ti plates can either be permanently retained in the body or removed through additional surgery after the fracture heals. During the bone-healing period of several months, the Ti plates maintain the stability of the fracture construct in the desired reduced position. However, if periprosthetic bacterial infections occur around the inserted implant, they can cause devastating complications such as early implant removal before bone healing, limb amputation, or even death [1,2]. Furthermore, the socioeconomic burden is highly increased for the treatment of patients with periprosthetic infections, which is approximately 7 times higher than those who are uninfected [3,4].

Therefore, the antibacterial efficacy of surgical implants has garnered remarkable attention [5]. The development of antibacterial surfaces for medical implants appears to be an extension of previous studies with a long-standing history of environmentally friendly antibiofouling surfaces for marine ships [6]. Typical strategies for creating antibiofouling surfaces include modifying the surface to prevent the adhesion of microorganisms and biofilm formation, creating contact-killing surfaces for microorganisms, and coating the surface with chemicals that release antibiofouling agents [7,8]. Among the numerous previous studies conducted, the excellent antibiofouling effect of the slippery liquid-infused porous surface (SLIPS), which has recently gained attention, has been demonstrated by applying a physical barrier that prevents microorganisms from settling [9,10]. Chae et al. [11] also demonstrated good efficacy through in vivo experiments using rabbits by applying the lubricated slippery surface to surgical implants composed of stainless steel [11].

However, previous studies on antibacterial surfaces for human use have certain limitations for practical application. First, while most materials used for creating antibacterial surfaces may be eco-friendly, their practical clinical application in the human body poses many regulatory hurdles for approval. For instance, polymers containing a substantial amount of fluorine, such as fluoroalkyl silane used in building construction materials such as concrete, are utilized for producing SLIPS. Second, previous studies often neglected to consider heat resistance during sterilization via autoclaving. Titanium plates used for bone fracture fixations are routinely autoclaved for sterilization, typically at 121 °C for 30 min with a 2.0-bar gauge, before being surgically implanted into the human body. Therefore, the heat stability of materials must be pragmatically assessed when fabricating metal implant surfaces for humans. In review of literature, we could not find any studies regarding the maintenance of antibacterial efficacy of titanium plates for orthopedic fracture surgery after autoclaving. Third, many prior studies utilized stainless steel, which is seldom used in modern metal implants [12,13]. Titanium is the most prevalent metal used for implants in contemporary medicine due to its superior strength, hardness, lightweight nature, stable corrosion resistance, biocompatibility, and nonmagnetic properties [14]. Fourth, numerous previous methods focused solely on creating nonadhesive surfaces to prevent biofilm formation on implant surfaces. However, unlike the sole concern for achieving an antibiofouling effect in the preparation of marine ship surfaces, medical implants must also possess an effective antibacterial effect within the surrounding host tissues. The interfaces between the implant and host tissues are susceptible to bacterial adhesion and biofilm formation. Biofilms settled in these interfaces prove challenging to eradicate with conventional serial irrigation and debridement procedures, which are standard surgical interventions used to eliminate biofilms from infection sites. Residual biofilms in specific interfaces can facilitate bacterial repopulation [15].

In this study, our team introduces a novel coating with a nanostructured surface layer on the Ti surface to provide sustained drug release. A thin nanostructured layer of Ti was fabricated to enhance the coating bond retention during the autoclave sterilization process and subsequent insertion into the body. While previous studies have suggested various materials for creating such a coating layer [16,17], this study focused specifically on existing Food Drug Administration (FDA)-approved materials. Three elements were chosen as proof of concept: poloxamer 407 (PLX; Pluronics), octanoic acid (OA; also known as caprylic acid), and epigallocatechin gallate (EGCG). PLX is approved for human use to prevent tissue adhesion during surgical injury [18] and is currently widely used in the medical polymer market [19]. During the optimization process of the metal coating, a nanostructured surface was applied to the plate to facilitate binding of the liquid coating to the surface. OA, a fatty acid found in biological membranes, was selected as one of the coating mixtures [20], because it has the lowest toxicity among fatty acids or bio-derived oils [21]. EGCG, a phytochemical, was chosen as a sustained-release substance due to its proposed antibacterial and antibiofilm effects against a broad range of microorganisms, including damaging bacterial membranes and inhibiting quorum sensing [22]. These selected materials also exhibited higher flash point temperatures, ensuring resistance to autoclaving conditions during sterilization [23].

The usefulness of Ti plates with nanostructures having sustained release of antibacterial effects, coated with PLX/OA/EGCG, was verified both in vitro and in vivo. As the proposed coating is antiadhesive, antibacterial, and immunonegative, the surface derived in this study was named ABINS (antibacterial immunonegative surface). Notably, an in vivo realistic rabbit surgical model was developed to resemble actual fracture surgical procedures in humans. The performance of the ABINS coating, which reduces bacterial adhesion on the Ti implant surface and surrounding host tissues while having low toxicity, is closely introduced. We propose that Ti medical metal with a nanosurface structure coated with ABINS is a novel alternative for surgical metal processing.

## Materials and Methods

### Surgical titanium plate coating

Ti (grade 23: 90% titanium, 6% aluminum, and 4% vanadium) plates measuring 15 mm × 24 mm with a thickness of 0.62 mm were procured from JEILMEDICAL Corporation, Korea. Hexane (96%) and NaOH (98%, bead form) were sourced from Samchun Chemicals (Seoul, Korea). EGCG, PLX, and OA were obtained from Sigma-Aldrich, Korea. Heptadecafluoro-1,1,2,2-tetrahydrodecyltrichlorosilane (HDFS) (JSI Silicone, Korea) and Krytox (DuPont, USA) were acquired from the market.

Initially, all Ti substrates underwent ultrasonic cleaning in acetone and deionized water using an ultrasonic washing machine (POWERSONIC 610, Hwashin Technology) for 15 min, followed by thorough drying in an oven (HQ-FDO 156, CORETECH, Republic of Korea) at 60 °C. The cleaned Ti plates were then immersed in a 0.5-mol concentration (0.5 M) NaOH solution and subjected to a temperature of 180 °C in an oven (HQ-VDO64, CORETECH, Republic of Korea) to induce nanostructure formation through oxidation in a hydrothermal synthesis autoclave reactor. Subsequently, the plates underwent 15 min of sonication and were dried in an oven at 40 °C.

For the fabrication of SLIPS, the Ti implants underwent fluorination in a mixture of *n*-hexane and HDFS at a volume ratio of 1,000:1 for 10 min, followed by drying in an oven at 60 °C for over 20 min. Subsequently, Krytox was applied to the Ti implants using a pipette and left in ambient air until the Ti surface was completely covered by Krytox.

In the preparation of ABINS, a mixed solution was prepared with a PLX:OA:EGCG weight ratio of 20:80:1; 10 g of PLX and 500 mg of EGCG were mixed and placed in an oven at 100 °C for approximately 30 min to liquefy. Because of the high viscosity of the mixed solution of PLX and EGCG, making it difficult to use for coating, 40 g of OA was added to dilute it. After applying the prepared mixture until it completely coated the surface of the Ti nanostructure, it was dried in an oven at 60 °C for over 48 h.

### Surface characterization

The surface structures of the plates were observed using high-resolution field-emission scanning electron microscopy (JEOL, Japan). The water contact angle (WCA) was measured and averaged at 5 different locations for each surface using a WCA analyzer (FemtoFAB, SmartDrop, Republic of Korea). To confirm whether the coating layer was properly formed, cross-sectional observation was performed using a focused ion beam (Hitachi, Japan) system.

### Biofilm formation and quantification

The bacteria used in this study were obtained from the National Culture Collection for Pathogens (NCCP; Korea), the national pathogen resources bank supporting research and development in preparedness, diagnosis, and therapy of infectious human diseases. *Pseudomonas aeruginosa* (NCCP 15783) and *Staphylococcus aureus* (NCCP 11489) were isolated from sputum and blood, respectively. The bacteria were cultured in tryptic soy broth unless otherwise specified. Biofilm formation on the Ti plates was conducted following a protocol described in our previous study [24], with minor modifications. The Ti plates were submerged in acetone and rinsed with ultrapure water, followed by autoclaving at 121 °C for 20 min at a pressure of 15 psi. After drying in an oven at 60 °C, the autoclaved Ti plate was partially submerged in a tube including bacteria culture to allow biofilm formation at the air–liquid interface in the middle. The caps were tightly closed during incubation at 37 °C while shaking at 50 rpm for 10 d [25]. Finally, the Ti plates were retrieved separately, and biofilm formation was quantified. Biofilms formed on the Ti plates were quantified by staining with crystal violet dye (CV; Sigma V5265, Sigma-Aldrich, USA) according to the following procedures [26]. After washing 3 times with autoclaved ultrapure water, the Ti plates were placed in a 0.1% CV solution for 10 to 15 min, followed by 3 additional washes with autoclaved ultrapure water. The CV-stained biofilms were air-dried and then dissolved in 30% acetic acid solution (Ducksan 414 extra pure grade, Korea) for 15 to 20 min. The solubilized fractions were transferred in triplicate (100 μl each) to 96-well plates, and their absorbances were assessed at 550 nm using an ultraviolet-visible spectrophotometer (Tecan Spark 10M Multimode Plate Reader, SparkControl) [27]. Data from repeated experiments (*N* = 2 to 4) were analyzed, and each experiment was performed in triplicate (*n* = 3).

### In vivo femur bone fracture model and induction of periprosthetic infection

This study was approved by the Ethics Committee of Hallym University Health Science Center, Korea.

A total of 24 New Zealand rabbits (4 months old, weighing 2.6 to 3.0 kg, male) were anesthetized with pentobarbital sodium (30 mg/kg). The rabbits were divided into 4 groups of 6 rabbits each. Group I served as the negative control, without any infection or treatment of the surgical plates (bare negative). Group II served as the positive control, with infection and bare plates (bare positive). Group III comprised Ti plates treated with previously lubricated SLIPS methods using Krytox [11], and group IV comprised Ti plates treated with ABINS methods.

Orthopedic implants specifically developed for animal long bone fractures were used in the current study (ARIX VET System bone plate, JEILMEDICAL Corporation, Seoul, Korea). The fixation system comprised variable-angled locking screw plates (145 mm in length, 6 mm in width, and 2.2 mm in thickness; rigid type) and locking screws (6 to 14 mm in length and 2.0 mm in diameter). The plates were cut with a cutting device provided by the implant company before use according to the length of each rabbit femur.

The process of setting up the model and fracture surgery protocols was thoroughly described in Supplementary Methods. The coated Ti plates were prepared with biofilms of *P. aeruginosa* (NCCP 15783) as described above, and additional drops of the bacterial suspension were applied directly on and adjacent around the plates on the day of surgery. After the experiment, all the animals were housed in single cages with sufficient water and food. The health status of all the animals, including activity, body weight, and body temperature, was assessed daily.

### X-ray analysis during the recovery period

Postoperative radiographs were obtained on the day of surgery for each subject to determine whether the femoral fracture fixation models were appropriately applied. Anteroposterior and lateral radiographs were taken. To analyze bone healing, follow-up radiographs were obtained from 3 randomly selected subjects per group at 4 and 8 weeks.

### Bone healing analyses with micro-computed tomography

Three animals in each group were sacrificed 4 weeks after surgery, and the remaining 3 animals in each group were sacrificed 8 weeks after surgery. At each sacrifice (4 and 8 weeks after surgery), micro-computed tomography (micro-CT) was performed on the femurs to quantify new bone formation, and gross photographs were obtained to check tissue adhesion and observe pus formation around the surgical sites. Micro-CT was performed as follows: The femurs were cleaned, fixed in 4% paraformaldehyde for 3 d, and dehydrated in 75% ethanol. The dehydrated bones were then scanned using micro-CT (SkyScan 1173, Bruker MicroCT, Kontich, Belgium) to generate 3-dimensional (3D) voxel images (2,240 pixels × 2,240 pixels) of the bone samples. An Al 1.0-mm filter was used to reduce the signal noise, and a high resolution (*E* = 130 kVp, *I* = 60A, and integration time = 500 ms) was applied to all the scans. The 3D volumes of the scanned samples were generated from the acquired 2D lateral projections using the NRecon software (version 1.7.4.6, Bruker microCT, Kontich, Belgium). For analysis, the 3D reconstructed images were segmented into cubes measuring 10 mm × 10 mm × 10 mm based on the fracture site. The callus volumes outside the cortical bone were then calculated.

The scanned bone volumes were digitally reoriented using the DataViewer software (version 1.5.1.2, Bruker microCT, Kontich, Belgium) and analyzed using the CT-Analyzer software (version 1.19.4.0, Bruker microCT, Kontich, Belgium). The relative x-ray absorption coefficients within the mature bone and callus were distinguished by their densities, and the callus volume was quantified.

### Bacterial infection and inflammation analyses with histology staining

For histological staining, the femur bones and surrounding tissues were isolated and fixed with 10% neutral-buffered formalin. The fixed tissues were dehydrated sequentially with ethanol (70%, 95%, and 100%) and xylene solution, followed by embedding in paraffin blocks. These blocks were sectioned to a width of 3 μm using a rotary microtome (Leica RM2255 Fully Automated Rotary Microtome). The sections were then deparaffinized and rehydrated by three 5-min immersions in xylene and subsequent immersion in a series of ethanol concentrations (100%, 95%, 90%, 80%, and 70%) before proceeding with the histological staining experiments.

For hematoxylin and eosin (H&E) staining, tissue-embedded slides were initially immersed in Mayer’s hematoxylin solution (HHM500, ScyTek) for 3 min, followed by rinsing in tap water for 2 min. During the bluing step, the tissues were incubated with a bluing reagent (BRT500, ScyTec) for 30 s and then rinsed with distilled water twice. Next, the cells were counterstained with eosin Y ethanol solution (EYB500, ScyTec) for 3 min, followed by rinsing with 95% ethanol. The slides were then dehydrated in a series of ethanol solutions, cleared in xylene, and mounted with a coverslip. Slide images were acquired using a slide scanner (Axio Scan.Z1, Carl Zeiss) with a ×20 objective lens and processed using the Zeiss ZEN slide scan software.

For Masson’s trichrome (MT) staining, slides were immersed in Bouin’s solution (BNF500, ScyTek) at 60 °C for 60 min in a fume hood and then rinsed in running tap water until the sections became completely clear. To differentiate the nuclei, equal parts of Weigert’s (A) (HWI-A-500, ScyTek) and Weigert’s (B) (HWI-B-500, ScyTek) solutions were mixed and used for staining the slides. After 4 min, the slides were washed under running tap water for 2 min. To stain the muscle fibers and erythrocytes, Biebrich Scarlet/Acid Fuchsin solution (BSU500, ScyTek) was applied to the slides for 4 min and washed with running tap water for 2 min. Subsequently, after differentiation in phosphomolybdic/phosphotungstic acid solution (PPA500, ScyTek) for 10 min, Aniline Blue solution (ABP500, ScyTek) was applied to the slides for 10 min to stain the collagen, followed by treatment with 1% acetic acid solution (AAE500, ScyTek) for 3 min. The slides were then dehydrated, cleared with ethanol and xylene, and mounted with a coverslip using a mounting solution. Slide images were obtained using a slide scanner as described above.

A HRP/DAB (horseradish peroxidase/3,3′-diaminobenzidine) detection immunohistochemistry (IHC) kit (ab64264, Abcam) was utilized for IHC, following the manufacturer’s protocol. Initially, the slides were immersed in prewarmed (95 °C) antigen retrieval solution (ab93678, Abcam) for 10 min and then allowed to cool to room temperature for approximately 30 min. Following a 5-min wash with distilled water, endogenous peroxidase activity was quenched with hydrogen peroxide for 15 min, followed by two 5-min washes with distilled water. Subsequently, the slides underwent permeabilization [0.05% Tween 20 in tris-buffered saline (TBST)] for 10 min, followed by blocking for 30 min. Primary antibodies were then applied and left to react overnight at 4 °C. The primary antibodies used were as follows: anti-interleukin-6 (IL-6) (ab6672, Abcam), anti-IL-1β (bs-6319R, Bioss Inc.), anti-tumor necrosis factor-α (TNF-α) (ab6671, Abcam), anti-CD11b (MCA802GA, Bio-Rad), and anti-neutrophil (MCA805GA, Bio-Rad). After being washed 3 to 4 times with TBST, the slides were incubated with biotinylated secondary antibodies for 30 min at room temperature. Following another wash cycle, the slides were incubated with streptavidin peroxidase for 30 min. Subsequently, the slides were rinsed 3 to 4 times with TBST and developed with DAB chromogen in the DAB substrate by incubation for 1 to 10 min. After immersion in distilled water twice for 2 min each, the sections were dehydrated, cleared with a series of ethanol and xylene solutions, and mounted with coverslips. Image analysis was conducted using a slide scanner, as described previously.

### Bacterial infection and inflammation analyses with Western blot

Fresh tissues surrounding the bone fracture were promptly collected upon euthanizing the rabbits and snap-frozen in liquid nitrogen. These frozen tissues were subsequently pulverized by grinding in a precooled mortar with liquid nitrogen. The resulting powdered tissues were dissolved in radioimmunoprecipitation assay buffer and incubated in a 95 °C heat block for 10 min. Protein extracts were then diluted with 1× phosphate-buffered saline, and the protein concentration was determined using the bicinchoninic acid assay. For SDS-polyacrylamide gel electrophoresis, the protein extracts were further diluted with 1× phosphate-buffered saline to ensure an equal amount of total protein (20 μg) per well. Protein electrophoresis with SDS-polyacrylamide gel electrophoresis was performed using the Hoefer apparatus, and gel transfer onto nitrocellulose membranes (0.2-μm pore; Bio-Rad) was carried out using a Bio-Rad Mini Trans-Blot Cell. The transferred membranes were blocked with 5% (w/v) of nonfat dry milk in 1× tris-buffered saline containing 0.1% Tween 20. Primary antibodies used included rabbit polyclonal anti-TNF-α (ab6671, Abcam), rabbit polyclonal anti-IL-1β (bs-6319R, Bioss Inc.), rabbit polyclonal anti-IL-4 (ab9622, Abcam), rabbit polyclonal anti-IL-6 (ab6672, Abcam), mouse monoclonal antibody against *P. aeruginosa* outer membrane protein (*P.A.* OMP) (clone B11, Thermo Fisher Scientific), and mouse monoclonal antibody against glyceraldehyde-3-phosphate dehydrogenase (GAPDH) as a control (clone G9, Santa Cruz Biotechnology). Secondary antibody reactions utilized HRP-conjugated goat anti-mouse immunoglobulin G (H+L) (31430, Invitrogen) and conjugated goat anti-rabbit immunoglobulin G (AP132P, EMD Millipore) antibodies. Blotting membranes with antibody binding were then incubated with enhanced chemiluminescence solution (SuperSignal West Pico PLUS Chemiluminescent Substrate, Thermo Fisher Scientific) and digitally documented using a chemiluminescence imaging system (WSE-6200 LuminoGraph II, ATTO Korea).

### Statistical analysis

Unpaired *t* tests were conducted using Prism 8 software (GraphPad Software Inc., USA). Data from the experimental groups were compared with the bare surface group as a control, and *P* values below 0.05 were considered significant. Statistical significance between groups is denoted in the graph figures or tables below the figures as follows: *P < 0.05, **P < 0.01, and ***P < 0.001; ns indicates no significant difference. One-way analysis of variance (ANOVA) was also performed using Prism 8 software to evaluate significant differences between the groups, with a significance level set at *P* < 0.0001.

## Results

### Overview of ABINS verification

**Fig. 1.**
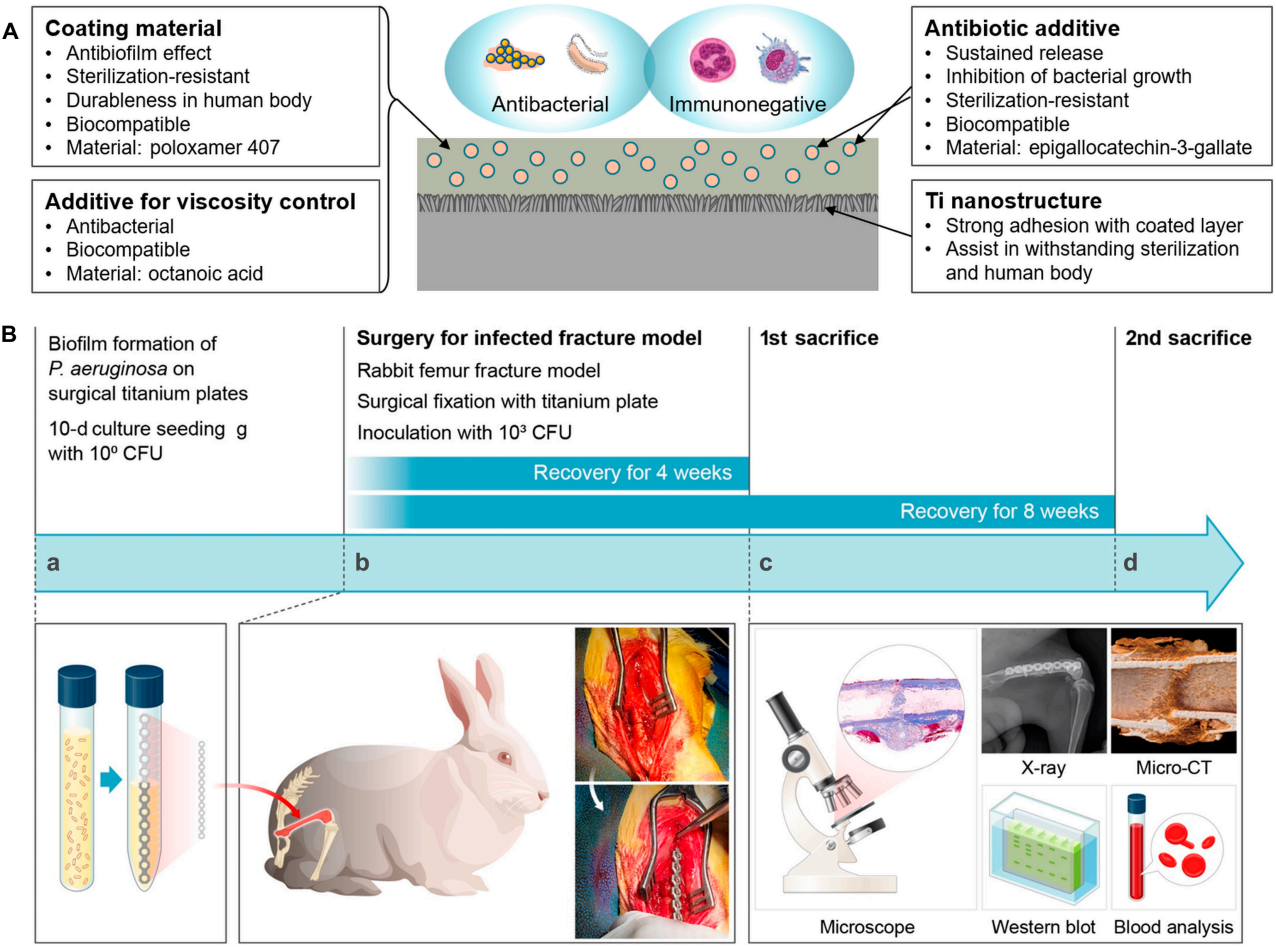
The composition and in vivo workflow of the ABINS. (A) Schematic diagram explaining the effects and roles of each element comprising ABINS. The Ti surface with nanostructure is layered with coating materials including PLX, OA, and EGCG. PLX serves as the primary coating material, while OA controls viscosity. EGCG is included in this coating layer as an antibiotic additive. The Ti nanostructure firmly holds the coating layer. (B) Overview of the in vivo processes and analysis plans for verifying the modified surface efficacy. The in vivo processes were largely divided into 4 steps (a to d): *P. aeruginosa* biofilm formation on surgical Ti plates (a), insertion of the coated Ti plate into the rabbit femur fracture model with additional bacterium inoculum (b), observation of recovery trends over the next 4 and 8 weeks, respectively, using various techniques (c and d).

The rationale and experimental procedures of this study are summarized in Fig. 1. Figure 1A illustrates the unmet needs of surgical metal plates and the novel functionalities of the metal coatings. In this study, antibacterial and biocompatible materials were selected on the basis of existing literature and our previous studies [24,28,29]. The primary coating material, PLX, offers antibiofilm and antiadhesion effects [30]. OA serves as an additive solvent to reduce the viscosity of PLX. OA, with the lowest toxicity among short-chain fatty acids naturally synthesized in human cells [20], also exhibits antibacterial effects [21]. EGCG was included to inhibit bacterial growth. Being amphipathic, EGCG can be gradually released from the oily phase into the surrounding aqueous environment (human tissues).

The entire process and experiments conducted to validate the in vivo efficacy of the metal coating are depicted in Fig. 1B. *P. aeruginosa* was chosen as the model bacterium for postsurgical infection. This bacterium is recognized as a important pathogen in postsurgical infections, with previous studies demonstrating its capability to form biofilms on metal plates [24,28–31]. Since addressing *P. aeruginosa* infection with biofilm formation poses a substantial challenge and has been [31,32] extensively researched, it can serve as a promising indicator for other bacteria if a metal coating can prevent *P. aeruginosa* infection. To better simulate bacterial infections during surgery, we utilized biofilm-formed surgical plates and applied additional bacterial inoculum to the implant site.

The in vivo fracture model was established using a modified veterinary surgery protocol, closely resembling a practical surgical model for humans, to ensure the formation of a complete fracture model. Specific surgical wires and screws were used for the fixation of Ti plates, effectively maintaining the fracture stabilization construct during the bone healing period, despite the active movement of rabbits during the study period. Following surgery, the Ti plates were evaluated at 4 weeks (first sacrifice) and 8 weeks (second sacrifice) using x-ray, micro-CT scans, blood tests, histology, and Western blot analyses. The results confirmed that the ABINS coating material demonstrated reduced bacterial adhesion to the implant surface and surrounding host tissues, as well as low adhesion and toxicity.

### Preparation of surfaces

**Fig. 2.**
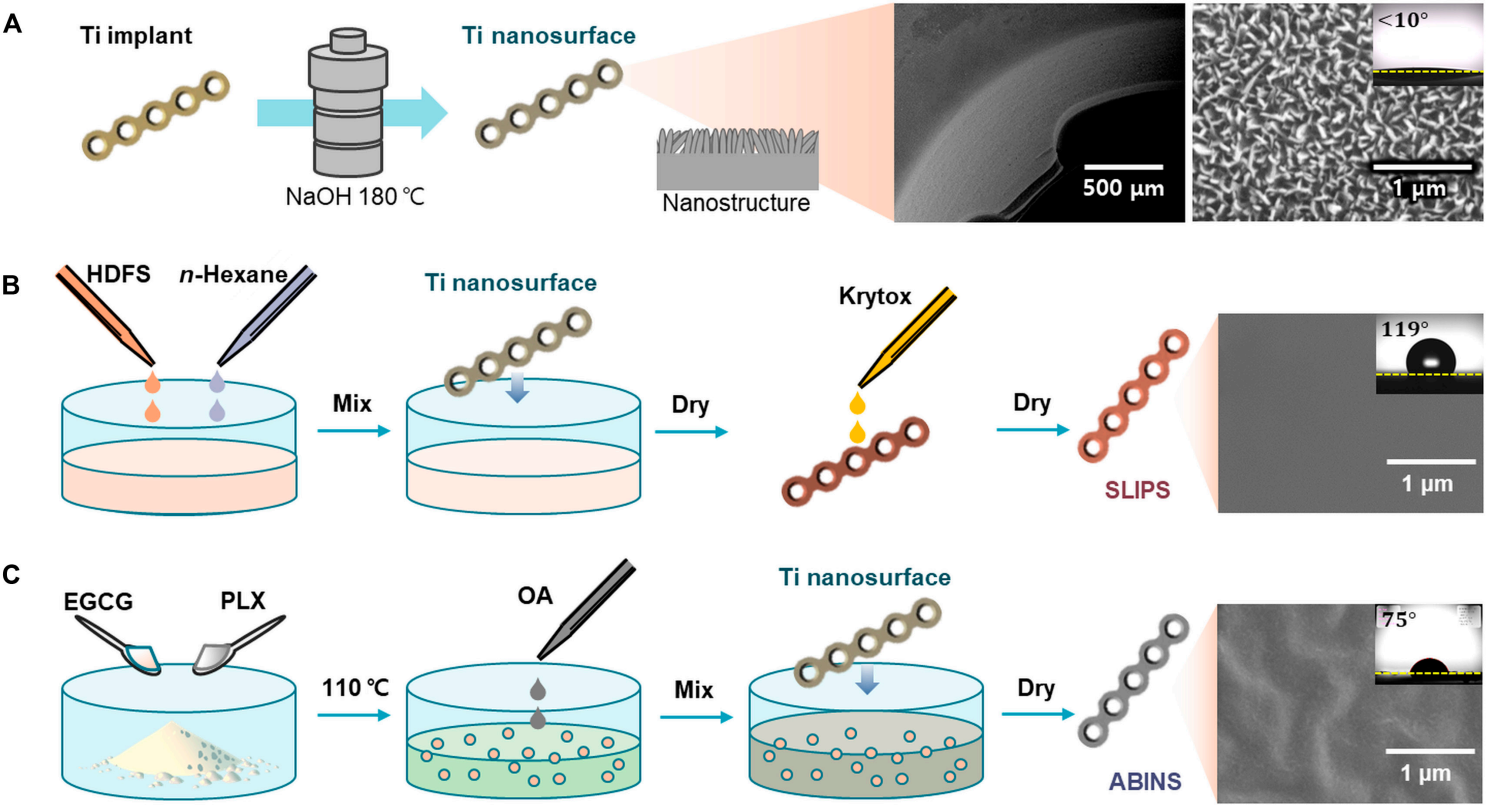
Conceptual diagram of Ti surface modification and scanning electron microscopy images showing surface structure with WCAs. (A) Nanostructure formation method on the Ti implant surface: The Ti implant is covered with nanostructures on a scale of approximately 10 to 20 nm. This surface structure, along with the hydrophilic properties of TiO_2_, exhibits superhydrophobic properties with a water droplet contact angle of less than 10°. The Ti implant with the nanostructure serves as the basis for the following 2 surface coating methods (SLIPS or ABINS). (B) SLIPS fabrication process: The nanostructured Ti surface undergoes coating in an HDFS-containing solution for 10 min, followed by a fluorination process, and then lubrication through Krytox coating to complete the SLIPS coating. The fabricated SLIPS surface demonstrates a smooth surface without roughness and hydrophobicity, with a contact angle of approximately 119°. (C) ABINS fabrication process: EGCG in powder form and PLX are evenly mixed and liquefied at a high temperature (110 °C). Subsequently, OA is additionally applied to the Ti implant surface. The ABINS surface exhibits wrinkles and a contact angle of approximately 75°.

To verify the efficacy of antibacterial surfaces for orthopedic bone fracture surgery, we fabricated SLIPS and ABINS on Ti plates. The surface modification process to impart the antibiofilm effect on Ti implants is illustrated in Fig. 2. A nanostructured surface was created using a sodium hydroxide aqueous solution as pretreatment to achieve SLIPS and ABINS coating on the Ti plate surface (Fig. 2A). The formation of TiO_2_ nanoscale (20 nm) structures involves dissolution and precipitation reactions.

Ti + 4H_2_O → Ti(OH)_3_^+^ + (OH) ^−^ + 2H_2_ (dissolution)

Ti(OH)_3_^+^ + OH^−^ → TiO_2_ + 2H_2_O (precipitation).

This surface enhances the capillary effect by incorporating nanostructures into the hydrophilicity of TiO_2_ and exhibits superhydrophilicity with a WCA of less than 10°. This nanoscale surface roughness contributes to robustly holding the SLIPS and ABINS coatings for subsequent applications.

Two candidate surface coatings for inhibiting biofilm formation were applied to a previously fabricated Ti nanostructure surface (Fig. 2B and C). First, the SLIPS was fabricated for comparison with the target coating material (Fig. 2B). A smooth surface is evident in the scanning electron microscopy image because Krytox, located at the outermost part of the SLIPS, covers all surfaces. The coated SLIPS exhibited an average WCA of approximately 119° and demonstrated surface properties that facilitated easy sliding of water droplets. Subsequently, the ABINS was prepared. A mixture of PLX, OA, and EGCG was coated onto a Ti nanosurface (Fig. 2C). When PLX is melted at 100 °C, it is obtained as a liquid but is not sufficiently diluted for coating; hence, it is necessary to add oil for dilution. Additives for dilution were investigated among biocompatible substances approved for human use; OA was selected and utilized, which can aid in biofilm suppression along with dilution (Fig. S1). It was confirmed through focused ion beam that the TiO_2_ oxide layer fabricated to form the nanostructure surface and the mixture of PLX, OA, and EGCG coating layer were formed (Fig. S2). The average WCA of the fabricated ABINS was approximately 75°, indicating weak hydrophilicity. Preservation of mechanical properties was also confirmed using a universal testing machine for maximal tensile strength for shear and bending stresses (Table and Fig. S3).

**Table.** Mechanical properties of the Ti implants for the shear force.

	Ultimate tensile strength (MPa)	Yield strength (MPa)	Elongation (%)	Young’s modulus (GPa)
Bare	580.77	400	21.77	19.089
Nano	576.92	397.435	20.17	21.410
SLIPS	578.85	403.846	20.65	20.633
ABINS	579.49	393.589	21.43	23.026

The developed ABINS fabrication method offers the advantage of utilizing Ti, a detail not extensively reported previously. Moreover, this straightforward process is beneficial for mass production and applicable to 3D surface shapes, thus advancing its industrial applicability.

### Evaluation of the antibiofilm effect

**Fig. 3.**
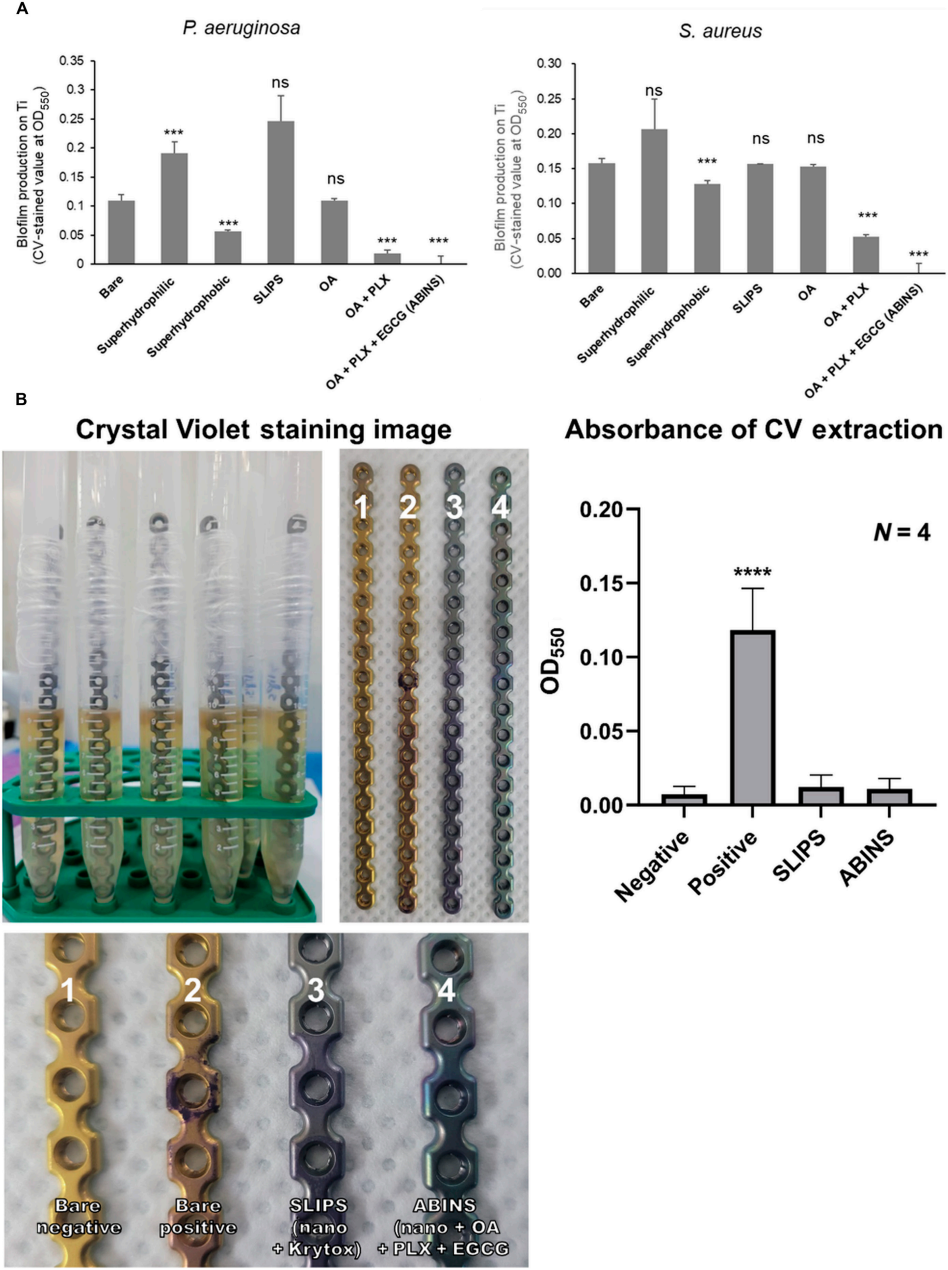
Evaluation of the antibiofilm effect of coated surfaces. (A) Effects of surface coating elements on biofilm formation: *P. aeruginosa* (NCCP 15783) and *S. aureus* (NCCP 11489) were induced to form biofilms on Ti plates coated with elements of SLIPS or ABINS, and biofilm formation was measured using a CV assay. The Ti plates were categorized as follows: bare, no structured and no coating Ti plate; superhydrophilic, nanostructured Ti plate with no coatings; superhydrophobic, nanostructured Ti plate with HDFS; SLIPS, nanostructured Ti plate with HDFS + Krytox; OA, PLX, and/or ABINS, nanostructured Ti plate coated with the indicated conditions. For both microorganisms, the coating materials for ABINS significantly inhibited biofilm formation on the Ti surface, indicating a promising outcome for in vivo applications. Statistical significance between the groups versus bare group was indicated as follows: ***P < 0.001. ns, no significant difference. (B) Biofilm formation testing with *P. aeruginosa* (NCCP 15783) on manufactured plates for in vivo experiments: Bare, SLIPS, and ABINS plates were incubated with *P. aeruginosa* (NCCP 15783) culture medium. Bare negative was used for no bacterial infection control. After 10 d, the amount of biofilm on each plate was evaluated by staining with CV solution. On the bare-negative plate, there were no stained sections observed, whereas in the bare-positive group, a wide stained section was visible in the middle of the plate. Stained sections were observed in the middle of both the SLIPS and ABINS plates, but they were smaller compared to those on the bare-positive plate. The absorbance value at optical density of 550 nm (OD_550_) measured by the CV assay is shown on the right panel. Each group is indicated as follows: 1, bare-negative group cultured with medium control without bacterial culture medium; 2, bare-positive group cultured with *P. aeruginosa* culture medium; 3, SLIPS group cultured with *P. aeruginosa* culture medium; 4, ABINS group cultured with *P. aeruginosa* culture medium. ****P < 0.0001.

Using rectangular Ti plates, a preliminary investigation was conducted to assess the impact of each element (nanostructure and coating materials) comprising the plate surfaces on *P. aeruginosa* and *S. aureus*, commonly found bacteria in biofilm-associated periprosthetic infections (Fig. 3A) [33]. For *P. aeruginosa*, the nanostructured superhydrophilic group exhibited a higher amount of CV-stained biofilm compared to the bare group. However, the superhydrophobic group, comprising the HDFS coating on the nanostructure group, significantly reduced biofilm formation, while the HDFS and Krytox coating on the nanostructure (SLIPS) group showed a comparable level of biofilm formation to the bare group. Conversely, the OA and PLX coatings on the nanostructure, or OA and PLX plus EGCG coatings on the nanostructure (ABINS) group, demonstrated potent inhibition of biofilm formation, although the OA-only coating on the nanostructure group yielded a similar level to the bare group. *S. aureus* exhibited a similar pattern to *P. aeruginosa*, except for the superhydrophilic nanostructure-only group, which did not significantly differ from the bare group. In the bacteria test, a statistically significant difference was observed among the OA, PLX, OA, and OA + PLX plus EGCG coating groups, indicating that the OA + PLX plus EGCG coating was most effective in inhibiting biofilm formation and constructing ABINS. In conclusion, the ABINS coating clearly demonstrated successful inhibition of biofilm formation on the Ti surface, presenting promising outcomes for in vivo applications.

Subsequently, the ABINS coating was applied to the Ti plates for an in vivo study, and its inhibitory effects on biofilm formation were confirmed by incubating with *P. aeruginosa* bacterial culture. Before surgery, CV staining of the preincubated plates was conducted, and only the bare metal plate with bacterial incubation demonstrated strong signs of biofilm formation. Plates with the SLIPS or ABINS coatings showed no biofilm formation upon CV staining (Fig. 3B).

### Assessment of biocompatibility

The coating materials for ABINS underwent further testing for biocompatibility by assessing cell adhesion to the coated surfaces using mouse fibroblast or macrophage cell lines (3T3-L1 or RAW264.7, respectively) directly seeded on the surfaces and cultured for 24 h (Fig. S4A). Compared to the bare surface, the OA + PLX coating decreased cell adhesion on the surfaces to 26% and 36% for fibroblasts and macrophages, respectively, while the OA + PLX plus EGCG coating reduced fibroblast or macrophage adhesion to 33% and 38%, respectively. Statistical analysis revealed that the results of the OA + PLX and OA + PLX plus EGCG groups were statistically significant compared to those of the bare control group, whereas there was no significant difference between the OA + PLX and OA + PLX plus EGCG groups. In summary, both the OA + PLX and OA + PLX plus EGCG coatings strongly inhibited cell adhesion to the surface.

Subsequently, it was investigated whether ABINS coating induced cytotoxicity by culturing cells in transwell inserts on conditioned surfaces. The results of the cell cytotoxicity assay demonstrated that both surface-coating conditions (OA + PLX and OA + PLX plus EGCG) maintained a cell viability of over 80% for both types of cells. As there was no statistically significant difference between the groups, the ABINS coating exhibited no serious toxic effects on the cells. In summary, the OA + PLX and OA + PLX plus EGCG coatings containing ABINS significantly reduced cell adhesion to the surface with no serious cytotoxicity (Fig. S4B and C).

### Application in rabbit fracture surgery model

The efficacy of the ABINS coating method was analyzed using an in vivo fracture surgery model (Fig. S5). A rabbit femur fracture model was adopted from previous studies [11,34], with further modifications to better simulate practical procedures for human fracture surgery. The detailed process and rationale for setting up a modified in vivo surgery model protocol are described in Supplementary Methods. Two modes of surgical infection were used to mimic surgical infections. The first mode involved biofilm formation on the implant, while the second involved opportunistic bacterial infection occurring during the surgical procedure. To simulate the former mode, we suggested a plate with a preformed biofilm with preincubation, as suggested in previous studies [11]. This approach facilitated effective periprosthetic infection by bacteria evading the rabbit’s immune system and antibiotic administration after surgery. To simulate the latter mode of infection, we administered additional bacterial inoculation using a bacterial culture solution with a concentration of 1 × 10^6^ colony-forming units (CFU)/ml; one droplet (1 μl) was pipetted directly onto the Ti plate, and another droplet (1 μl) was pipetted onto the adjacent tissue near the plate [35].

### Assessment of bacterial infection and inflammation

**Fig. 4.**
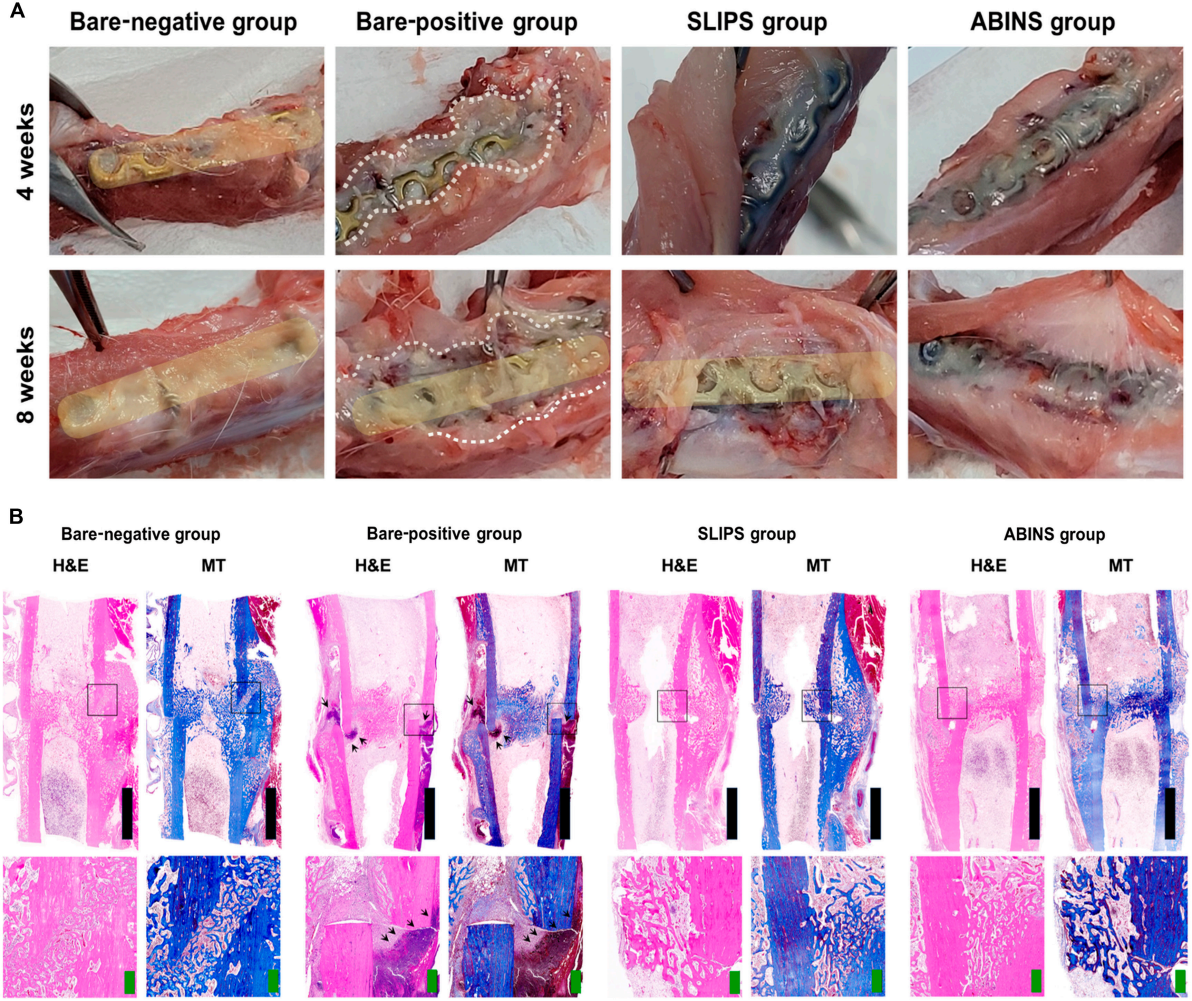
Assessment of bacterial infection and inflammation by gross examination and histological analysis: H&E and MT staining. (A) Gross examination of the surgical site: At 4 weeks, in bare-negative group, titanium plate (yellow rounded rectangle) was partially covered with tissue adhesion formation; in bare-positive group, infectious necrotic pus (white dashed line) with partial tissue adhesion formation over the titanium plate were observed; in SLIPS and ABINS groups, clear noninfectious surrounding tissues and minimal tissue adhesion formations were observed. At 8 weeks, in bare-negative group, titanium plate was almost obscured by severe adhesion tissue formation; in bare-positive group, infectious necrotic materials were observed around titanium plate; in SLIPS group, infectious tissues were not observed, but titanium plate was partially covered with tissue adhesion formation at both ends; in ABINS group, infectious tissues were not observed, and adhesion tissue formation was minimal. (B) H&E and MT staining: Tissue samples from the 4-week sacrifice group were stained with H&E and MT staining solutions. In the bare-negative, SLIPS, and ABINS groups, sparse immunological reactions were observed (indicated with the black arrows). However, in the bare-positive group, inflammations of the fractured bones were evident. The top images depict overall stained tissue images, while the bottom images show magnified views. Scale bars, 5 mm (black) and 0.5 mm (green).

Three rabbits per group were sacrificed and investigated at 2 time points: 4 and 8 weeks after surgery. Figure 4 presents the gross structural and histological analyses around the surgical site, signs of infection, and immune reactions. Figure 4A demonstrates that the positive control (bare metal plate with bacterial biofilm) exhibited pus around the surgical site in both the 4- and 8-week sacrifice groups, indicating a well-established bacterial infection in the femoral fracture/metal implant model of this study. No signs of infection were observed in the negative control (bare metal plate without bacterial preincubation). The Krytox-coated SLIPS plate, another negative control, also showed no infection at the surgical site. Remarkably, the group with the ABINS coating displayed no signs of infection and was indistinguishable from both negative control groups.

**Fig. 5.**
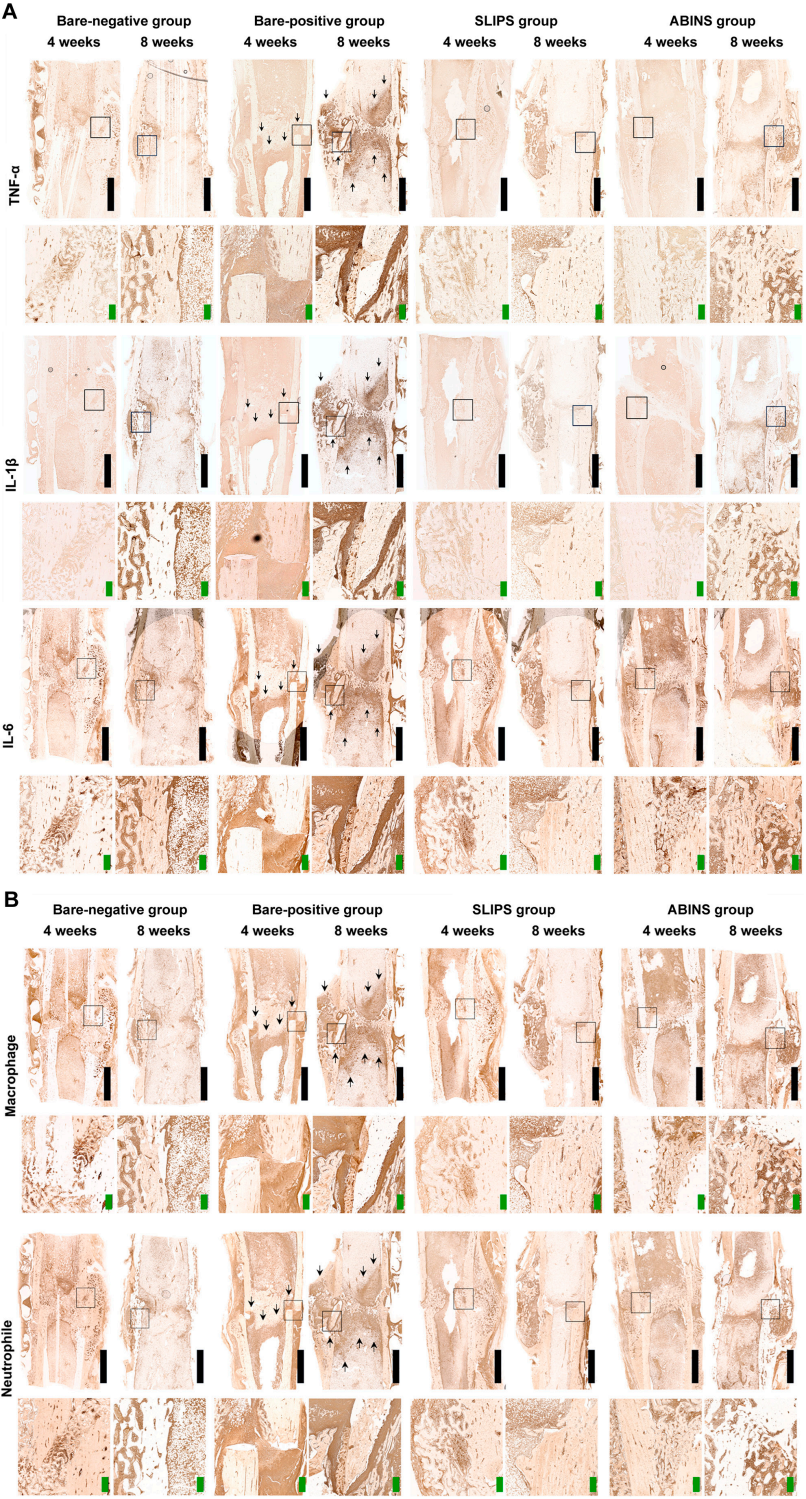
Assessment of bacterial infection and inflammation by IHC. Rabbit femur-surrounding tissues from each experimental group were assessed for inflammatory immune responses by detecting (A) inflammatory cytokines IL-6, IL-1β, and TNF-α and (B) inflammatory immune cell infiltration such as macrophages and neutrophils. In the bare-negative, SLIPS, and ABINS groups, minimal immunological reactions were observed. However, the bare-positive group exhibited pronounced immunological reactions, indicated by positive brown-color staining around the surgical site (the regions are indicated with the black arrows). Scale bars, 5 mm (black) and 0.5 mm (green).

Both H&E and MT staining (Fig. 4B and Fig. S6) revealed the infiltration of immune cells and inflammation of fractured bones in the positive group. This immunological reaction was barely observable in the negative control and experimental groups treated with the ABINS coating. IHC (Fig. 5) also confirmed that the inflammatory cytokines such as TNF-α, IL-1β, and IL-6 (Fig. 5A), as well as infiltration of inflammatory immune cells including macrophages and neutrophils (Fig. 5B), were detected only in the positive control group. Similar results were obtained in the 4- and 8-week sacrifice groups. This suggests that the developed surgical metal plate with a nanostructure and ABINS coating is free of biofilm and immunologically safe.

**Fig. 6.**
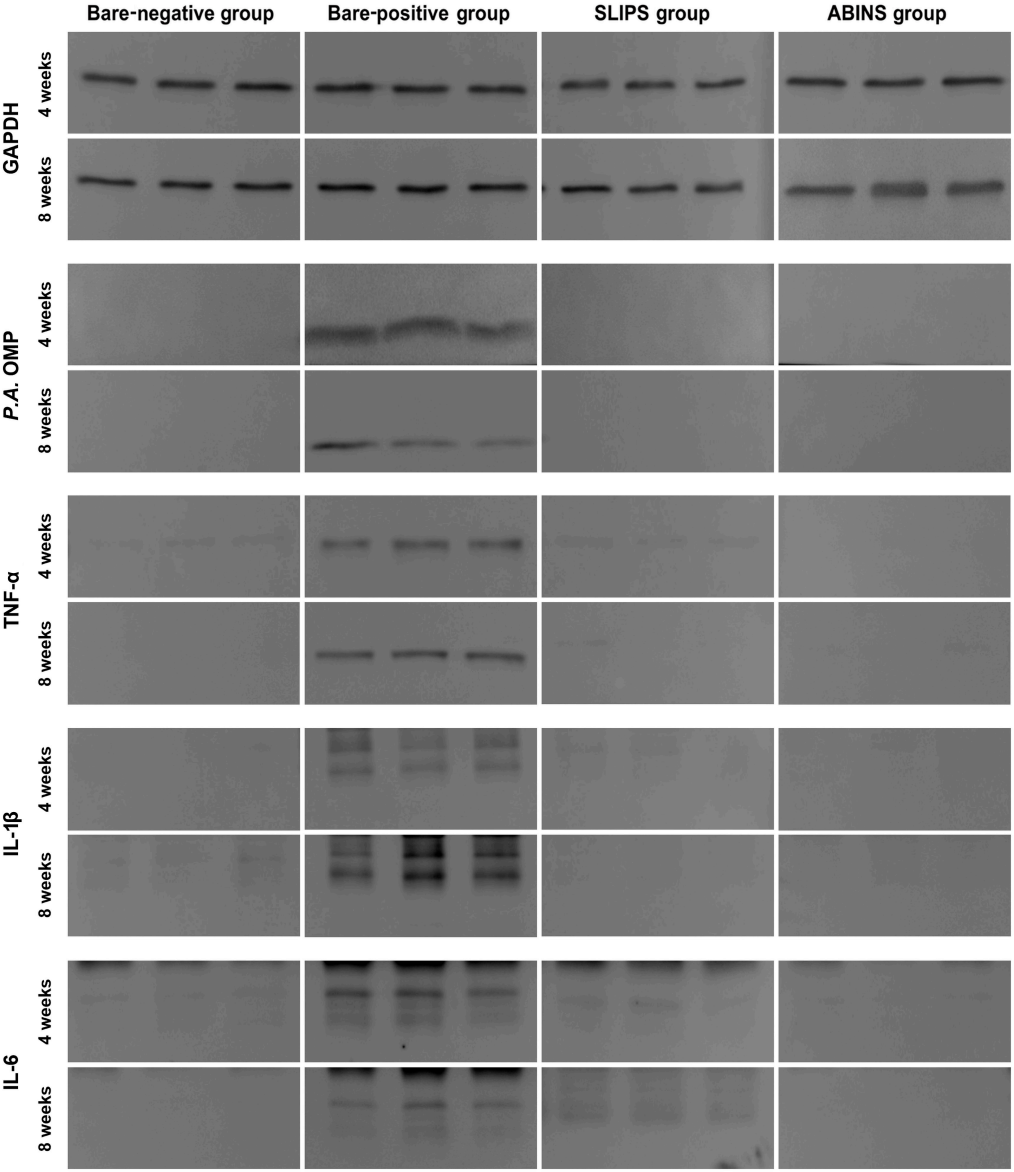
Assessment of bacterial infection and inflammation by Western blot. Western blots were conducted using rabbit biopsies around the surgical sites for the *P.A.* OMP, TNF-α, IL-1β, and IL-6. GAPDH was utilized as an internal control. Clear bands were observed only in the bare-positive group, indicating reaction with the *P. aeruginosa*-specific antibody, *P.A.* OMP, and antibodies against the inflammatory cytokines. In contrast, no reactions with the *P. aeruginosa* antibody were detected in the bare-negative, SLIPS, and ABINS groups. Similarly, other markers for the inflammatory cytokines were also detected exclusively in the bare-positive group.

To investigate bacterial infections in host tissues around the surgical site, as well as infections caused by the pathogens used in this study, we performed Western blotting using tissue biopsies around the surgical sites. Figure 6 demonstrates that the positive control group tested positive for the *P.A.* OMP marker, indicating *P. aeruginosa* infection occurred in the bare-positive group. In addition, the bare-positive control group showed major cytokines, including TNF-α, IL-1β, IL-4, and IL-6. The Western blot results of the groups corresponded to gross observations and histological images demonstrating immune cell infiltration. In conclusion, the Western blot results clearly show that the metal nanostructure with ABINS is safe for the body and possesses antibacterial abilities comparable to those of SLIPS.

### Radiological bone healing assessment

**Fig. 7.**
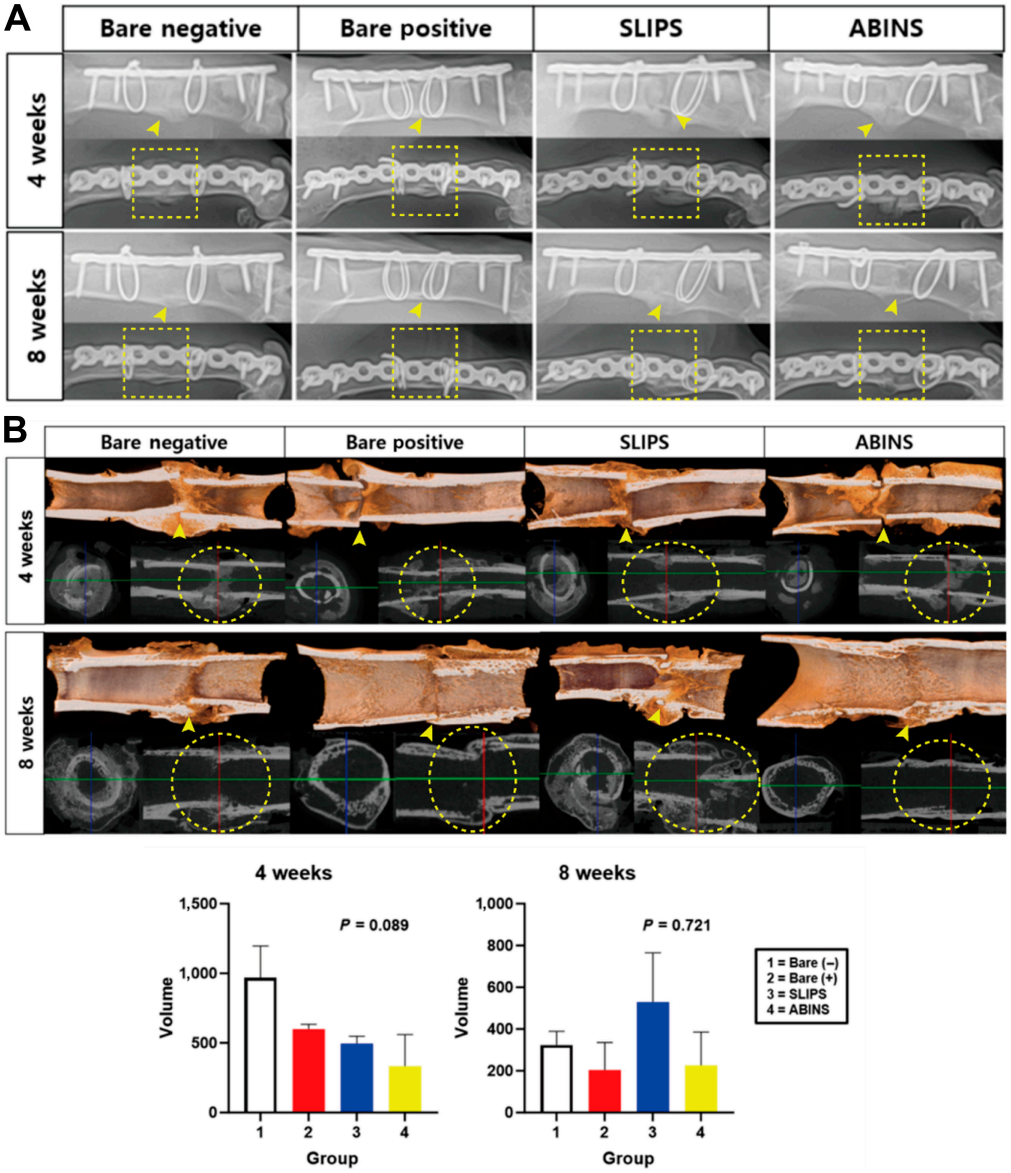
Radiological assessment of bone healing. (A) Anteroposterior and lateral radiographs were captured for each subject at 4 and 8 weeks after surgery. Initial fracture lines were visible between the 2 metal band wiring areas (arrow heads). At 4 weeks, increasing bone calluses were observed with callus bridges forming, and then fractures were completely healed by 8 weeks (dashed box). It is worth noting that the amount of calluses decreased because of fracture healing remodeling at 8 weeks. (B) Micro-CT images obtained at 4 and 8 weeks after surgery revealed that the volume of new callus formation did not exhibit significant differences among the 4 groups at either time point, with *P* values of 0.089 and 0.721, respectively. The arrow heads indicate initial fracture site, and dashed circles indicate healing bony calluses.

Figure 7 presents the radiographic analyses of bone healing and blood test results. Every subject demonstrated increased callus formation at 4 weeks after surgery, and complete bone healing was observed at 8 weeks after surgery (Fig. 7A and Fig. S7). Micro-CT analysis revealed no significant differences in the volume of new callus formation among the 4 groups at both 4 and 8 weeks (Fig. 7B). Osteoclastic activity was confirmed through tartrate-resistant acid phosphatase staining (Fig. S8), and the distribution of osteoblasts was assessed via immunofluorescence staining for alkaline phosphatase (Fig. S8).

The serum creatinine, aspartate aminotransferase, alanine aminotransferase, and C-reactive protein levels showed no significant differences among the 4 groups at 4 weeks after surgery (*P* = 0.869, 0.923, 0.345, and 0.078, respectively; Fig. S9A) and at 8 weeks after surgery (*P* = 0.459, 0.636, 0.243, and 0.982, respectively; Fig. S9B). In summary, none of the surgical plates caused systemic problems in the in vivo models.

## Discussion

Periprosthetic infections subsequent to surgical treatment of bone fractures not only contribute to the emergence of multidrug-resistant bacteria due to prolonged antibiotic use but also markedly incapacitate patients, leading to issues such as nonunion of fractures, early implant removal before bone healing, impaired joint functions, joint stiffness, limb amputation, prolonged hospital stays, or even death [1,2].

Most strategies outlined in previous literature face limitations in practical application to the human body. One reason for this is that many prior methods primarily offer antibiofouling effects on metal surfaces, drawing inspiration from antibiofouling surfaces found on marine ships. However, in clinical scenarios, biofilm formation in multiple niches between host tissue and implanted devices can be more challenging to eradicate. Bacterial infections anatomically localize in various areas, including overlying tissue fluid, foreign bodies (implant surfaces), and host tissue surfaces [15]. Conventional serial washing and debridement surgical procedures often fail to eliminate bacterial colonies or biofilms settled in these host tissue niches. Remaining biofilms in such niches serve as sources of pathogens [15]. Therefore, for practical application, an effective strategy for antibacterial surfaces for medical implants should encompass dual actions: prevention of bacterial adhesion on the implant surface and in the vicinity of host biological tissues.

Previous literature also noted that poloxamer can be used as solubilizer for antibacterial compounds. Antibacterial drugs like chloramphenicol [36], melatonin [37], or boron-containing [38] hydrogel were used in poloxamer gels for antibacterial skin dressing for wound healing agents. Recently, An et al. [39] used poloxamer gels (PLX 407 and PLX 188) with propolis to test its antifungal and antibacterial function. Their compound showed good antibacterial and anti-fungal activity for *S. aureus* and *Candida albicans*, respectively. Most of previous literatures used poloxamer gel for burn or diabetic ulcer skin dressing materials for antibacterial function against planktonic bacteria using amphiphilic structure and thermophilic gelation properties of poloxamer. Ouyang et al. [40] reported the outcomes of their pursuit of an antibiofilm effect by incorporating Ag into the surface of a SLIPS base. While silver nanoparticles (AgNPs) are widely recognized for their potent antimicrobial activities [41], concerns persist regarding the release of AgNPs and associated adverse effects and toxicity [42]. Released Ag^+^ ions from AgNPs have been shown to be locally delivered to cells at high [43] concentrations, potentially causing mitochondrial and DNA damage [38] and developmental defects [44]. Furthermore, both AgNPs and Ag ions can generate reactive oxygen species and induce oxidative stress in cells, thereby triggering inflammatory responses [45]. Kratochvil et al. [46] demonstrated the effectiveness of incorporating gradually releasing small-molecule quorum sensing inhibitors into SLIPS, showing promise in combating biofilm formation. A research group tested the SLIPS concept using stainless steel plates in vivo, revealing its applicability in bone fracture surgery [11]. However, this method was only feasible with small plates, limiting mass productivity. Moreover, recent bone surgeries have favored Ti instead of over stainless steel plates due to its superior mechanical strength [14]. Consequently, to develop an antibacterial coating for metals, a medical-grade Ti plate with FDA-approved coating materials becomes imperative. In particular, the antibacterial activity of EGCG-incorporated compositions has recently been reported in diverse fields with state-of-art techniques such as nanofiber, polymer film, 3D scaffolding, etc. [47,48]. This rationale led to the adoption of the ABINS coating in this study.

The ABINS coating technique used in this research holds promise in mitigating postsurgical issues associated with orthopedic implants [49]. The in vivo model demonstrated robust antibacterial and immune-evasive effects of the ABINS plates. Histological staining and Western blotting results indicated no evidence of inflammatory reactions or bacterial infections on the ABINS plate. These advantageous effects are comparable to those observed with another coating technique, the SLIPS plate with a perfluorinated chemical coating surface.

The coating developed in this study offers notable advantages, as the materials (OA and PLX) are FDA-approved safe materials suitable for surgical use. In addition, it has the capability to deliver various drugs, such as EGCG.

The effectiveness of the plate coating in reducing biofilm formation of *S. aureus* and *P. aeruginosa* in vitro, as well as in preventing periprosthetic infections of *P. aeruginosa* in vivo, was confirmed. The in vivo results regarding the antibacterial effect on *P. aeruginosa* were comparable to those of previous studies using SLIPS methods with *S. aureus* [11,50]. *P. aeruginosa* was selected as the model bacterium due to its greater capability in forming biofilms compared to *S. aureus* [24]. On the basis of the in vitro bacterial assay for *S. aureus*, surface preparation with an ABINS coating is expected to be effective in preventing periprosthetic infection by *S. aureus* after surgery.

In the future, the use of another antibacterial agent instead of EGCG is likely to yield similar effects. Moreover, other functions, such as promoting wound healing and improving bone formation, may be achievable when using different sustained-release drugs and EGCG. Given that PLXs are amphiphilic and OA is hydrophobic, drugs with various properties are anticipated to be utilized in future applications.

The rabbit femur fracture model experiments also demonstrated the safety of ABINS in vivo. There were no notable issues observed in the blood tests of the rabbits in the experimental group, and they remained healthy throughout the experimental period (8 weeks). Notably, the experimental period extended longer than that of previous in vivo studies, reducing biosafety concerns compared to other tested coating materials. Biomarkers such as TNF-α, IL-1β, and IL-6 were utilized to assess the inflammatory response; however, their expression levels were similar to those of the negative control group. Therefore, ABINS is suggested to possess immune-evasive properties as well.

In conclusion, we propose that ABINS will facilitate the development of more useful Ti plates in clinical settings.

## Data Availability

The Supplementary Materials include detailed experimental procedures, H&E, MT, tartrate-resistant acid phosphatase staining, and biofilm effect (PDF).
