# Supplementary material for: Antibacterial Immunonegative Coating with Biocompatible Materials on a Nanostructured Titanium Plate for Orthopedic Bone Fracture Surgery
Source: Biomater Res. 2024 Sep 11;28:0070. doi: 10.34133/bmr.0070 (PMC11387750; doi:10.34133/bmr.0070)
Supplement: Supplementary 1 — Figs. S1 to S9 Supplementary Methods [file bmr.0070.f1.docx]

**Supporting Information**

**Antibacterial immunonegative coating with biocompatible materials on a nanostructured titanium plate for orthopedic bone fracture surgery**

Jeong-Won Lee^1, †^, Jung-Ah Cho^2,3, †^, Yoo Jin Roh^4, †^, Min Ae Han^4,5, †^, Je-Un Jeong^1^, Sivakumar Allur Subramanian^2^, Eun-ho Kang^4^, Jiwoo Yeom^4^, Chang-Hun Lee^4,5*^, Sung Jae Kim^2*^

^1^Department of Mechanical Engineering, Chosun University, Gwangju 61452, Republic of Korea

^2^Department of Orthopedic Surgery, Dongtan Sacred Hospital, Hallym University, Hwaseong, Republic of Korea

^3^College of Transdisciplinary Studies, School of Undergraduate Studies, Daegu Gyeongbuk Institute of Science and Technology, Daegu 42988, Republic of Korea

^4^Department of New Biology, Daegu Gyeongbuk Institute of Science and Technology, Daegu 42988, Republic of Korea

^5^New Biology Research Center, Daegu Gyeongbuk Institute of Science and Technology, Daegu 42988, Republic of Korea

^†^: Authors contributed equally

*Corresponding authors:

Sung Jae Kim: sung1383@hanmail.net,

Chang-Hun Lee: leech@dgist.ac.kr

Supplementary figure 1


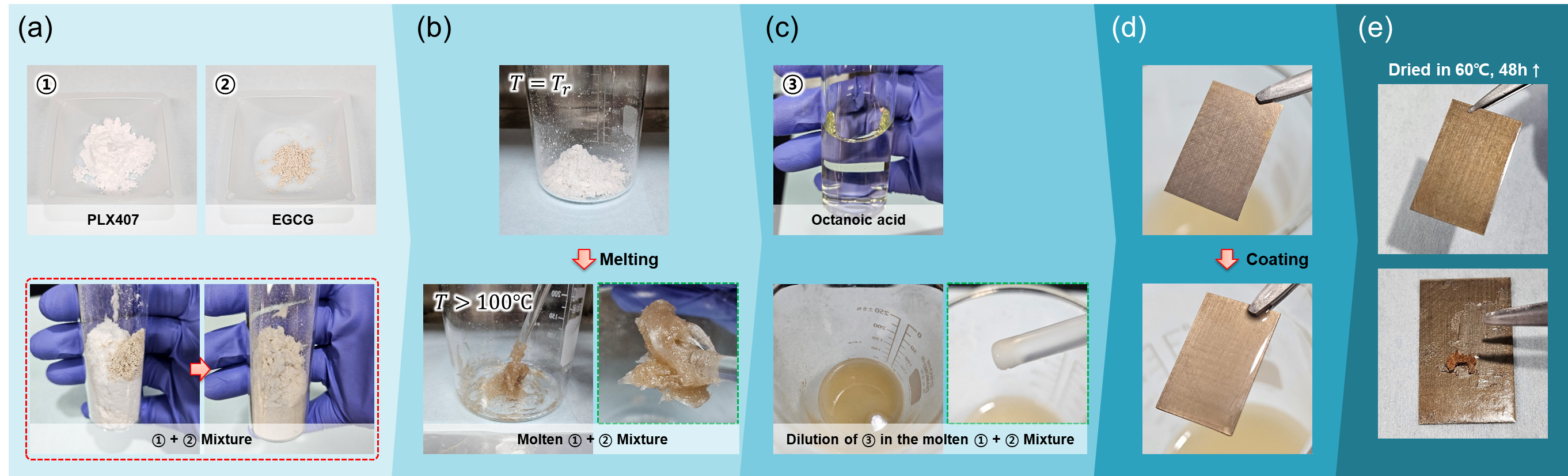


**Supplementary Figure 1.** Diagram showing the ABINS surface modification process with photos. (a) 10 g of PLX and 500 mg of EGCG are placed in a bottle and mixed evenly. (b) The mixture melts and gels above 100℃. The gelled mixture has a very high viscosity and tends to stick to the glass rod and clump together. Coating is impossible in this condition. (c) When 40g of octanoic acid is mixed into the galled mixture at high temperature, it becomes a dilute yellow liquid. Coating becomes possible at this stage. (d) When the TiAl6V4 plate with the nanostructure formed is immersed and taken out, the surface is evenly coated. (e) TiAl6V4 plate completely dried in a drying oven at 60℃ shows a transparent and glossy solidified coating surface. Due to a texture similar to solid wax, it does not show adhesion in the tape peeling test.

Supplementary figure 2


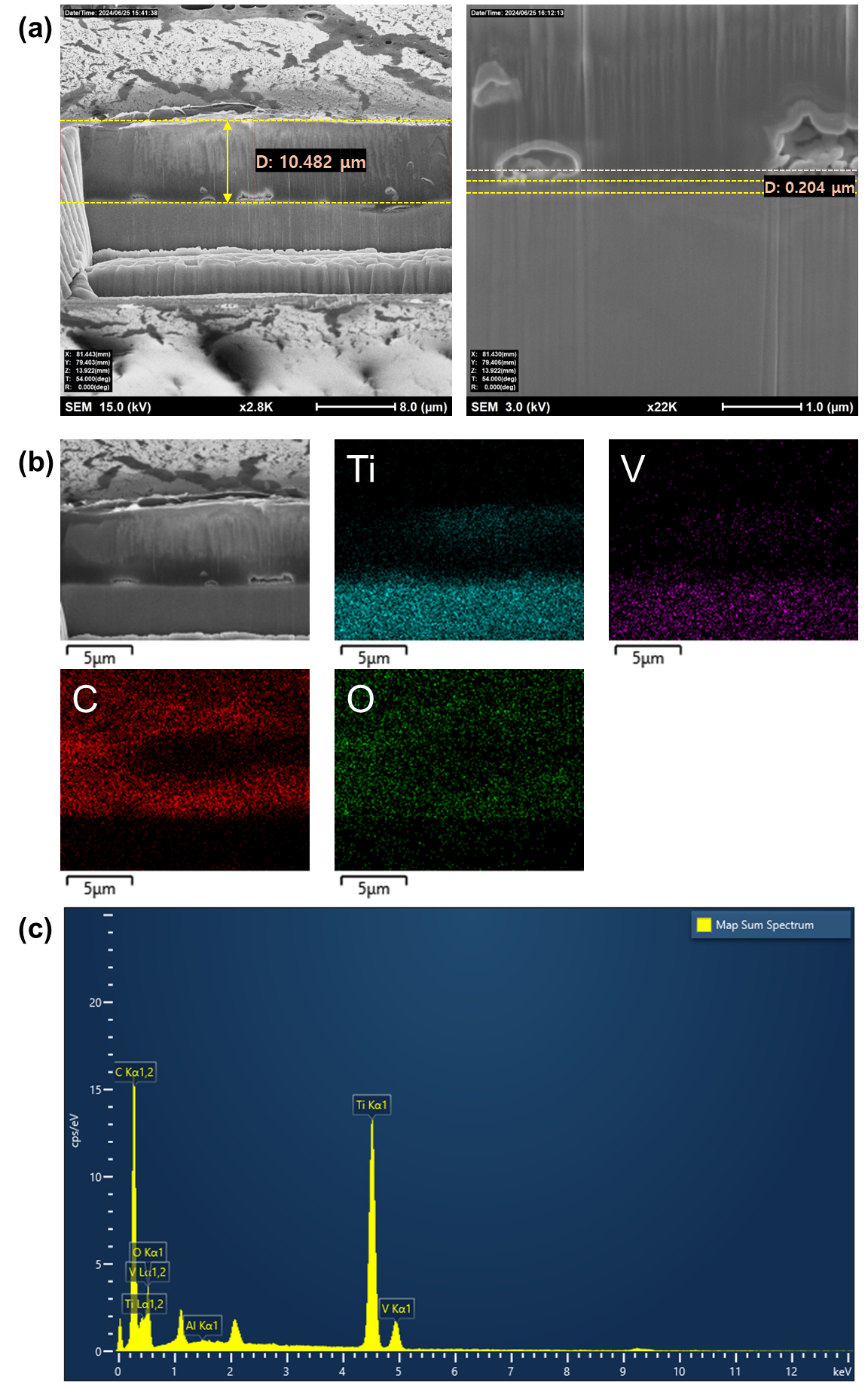


**Supplementary Figure 2.** ABINS cross-section data observed using the Focused Ion Beam (FIB) technique. (a) It is observed that a TiO_2_ oxide film is formed on the surface of the Ti implant with a thickness of about 200-300 nm, and an OA+PLX+EGCG layer is formed about 10 μm on top of it. (b) From the element mapping results, it is confirmed that the boundary between the plate part where Ti and V are detected and the coating part where C and O are detected is clearly divided. (c) All surface detected elements were Ti, V, O, and C as expected. Aluminum, one of the elements of TiAl6V4, is not detected in significant amounts, around 1%.

Supplementary figure 3

|  | Maximal load (N) | Stiffness (N/mm) |
| --- | --- | --- |
| Bare negative control | 442.2 | 124.4 |
| SLIPS | 440.9 | 125.2 |
| ABINS | 428.8 | 116.6 |


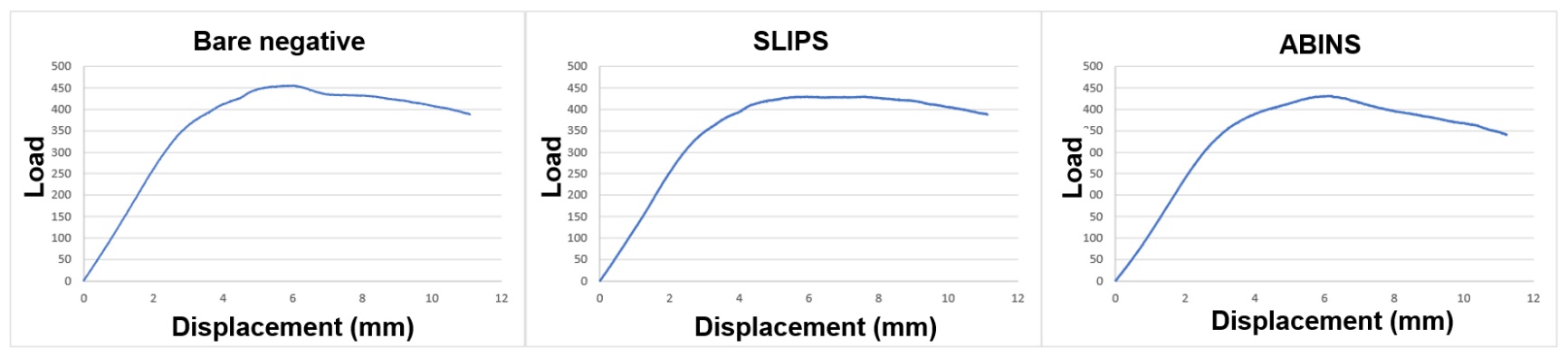


**Supplementary Figure 3.** Summary of stress test results for bending torque and load displacement curve of each plate preparation. The preservation of mechanical properties was confirmed universal testing machine for maximal tensile strength for shear and bending stress.

Supplementary figure 4


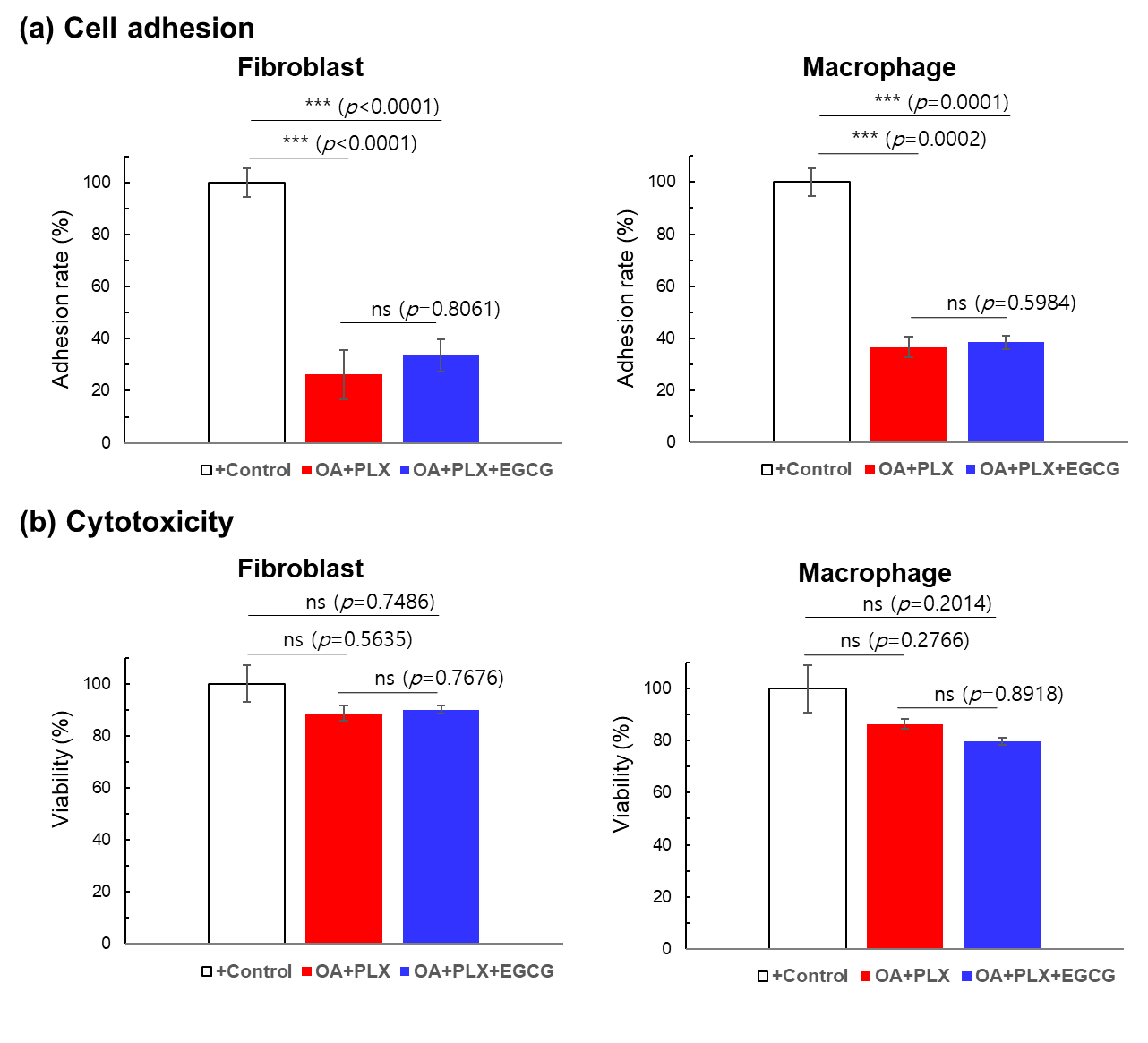


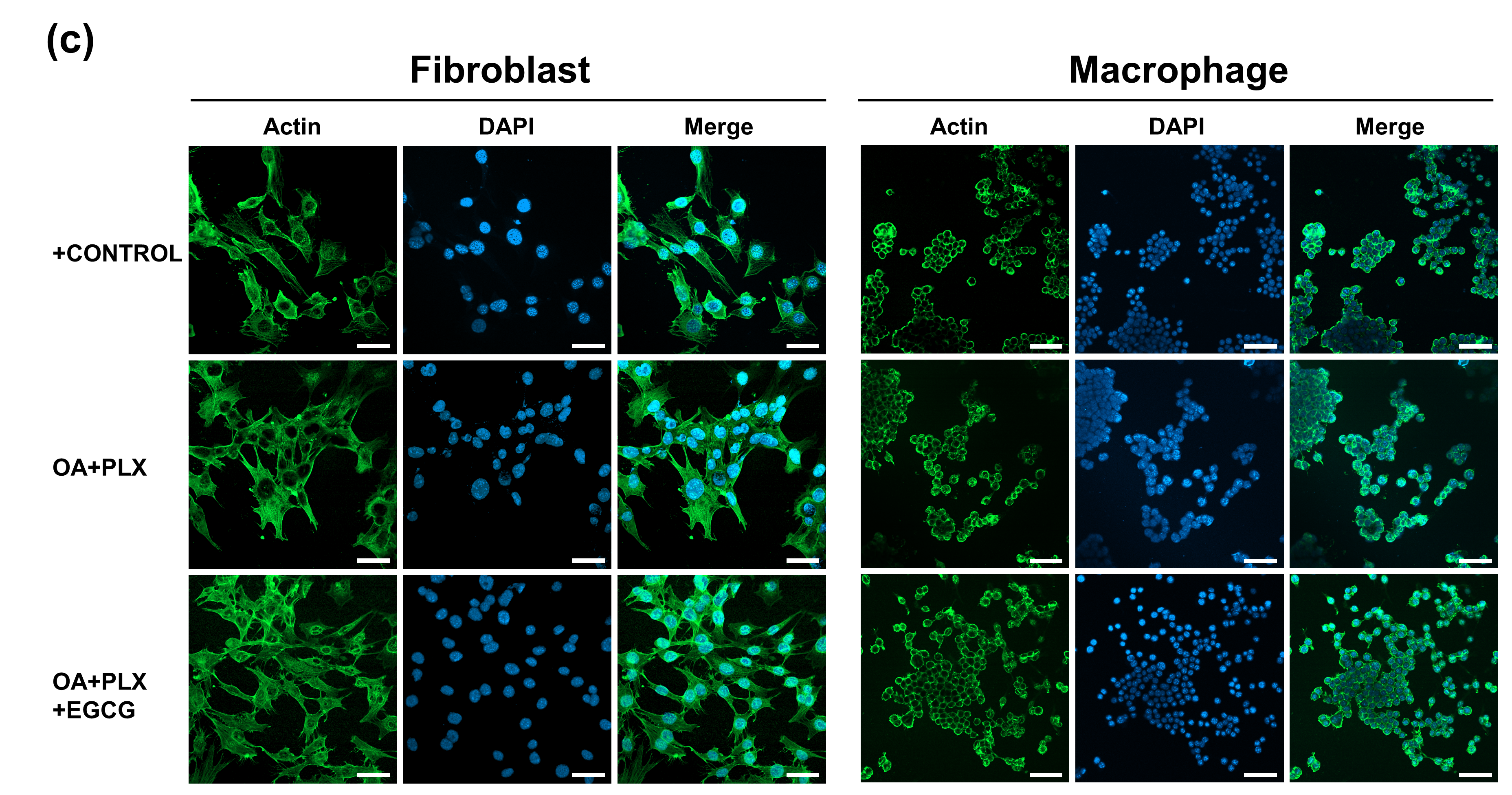


**Supplementary Figure 4.** Evaluation of the anti-adhesion effect and cytotoxicity of the coating materials.. Each Ti plate was modified and coated with specific conditions; bare surface ((+) control), poloxamer (OA+PLX), or poloxamer plus EGCG (OA+PLX+EGCG) group. Fibroblast and macrophage cells were cultured in the presence of each Ti plate. They were represented as different color bars. (white= (+) control, red= OA+PLX, blue= OA+PLX+EGCG) The upper graphs (a) show mean ± SD value of the adhesion rate and the lower graphs (b) show cytotoxicity (%). The values of adhesion rate and cytotoxicity were obtained from the CCK-8 assay by calculating their optical density OD 450 nm. The coating materials for ABINS (OA, PLX, EGCG) significantly reduced the cell attachment onto the surface with no serious cytotoxicity. The statistical significance between groups was indicated as follows; *** for P < 0.001, and ns for no significant difference. (c) The effects of the metal coatings on cell morphologies were evaluated using immune-fluorescence staining of actin with confocal microscopy. The cells cultured with the metal plates of the poloxamer coating (OA+PLX) or poloxamer plus EGCG (OA+PLX+EGCG; ABINS) were indistinguishable from the cells with the bare surface metal ((+) control)). Actin staining (Green); Nucleus (DAPI; Blue); Scale bars (White: 50 μm).

Supplementary figure 5


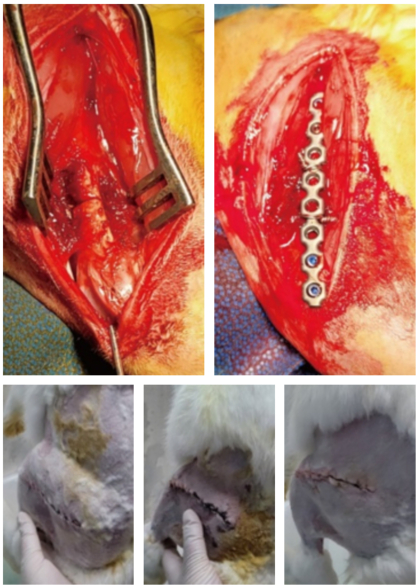


**Supplementary Figure 5.** Implantation of the Ti surgical plates for in vivo studies. The photos show the rabbit femur fracture surgery models. The surgical plates incubated with the same condition were implanted unto the fractured bones of the models. All the rabbits after surgery including positive control group showed no sign of unhealthy condition.

Supplementary figure 6


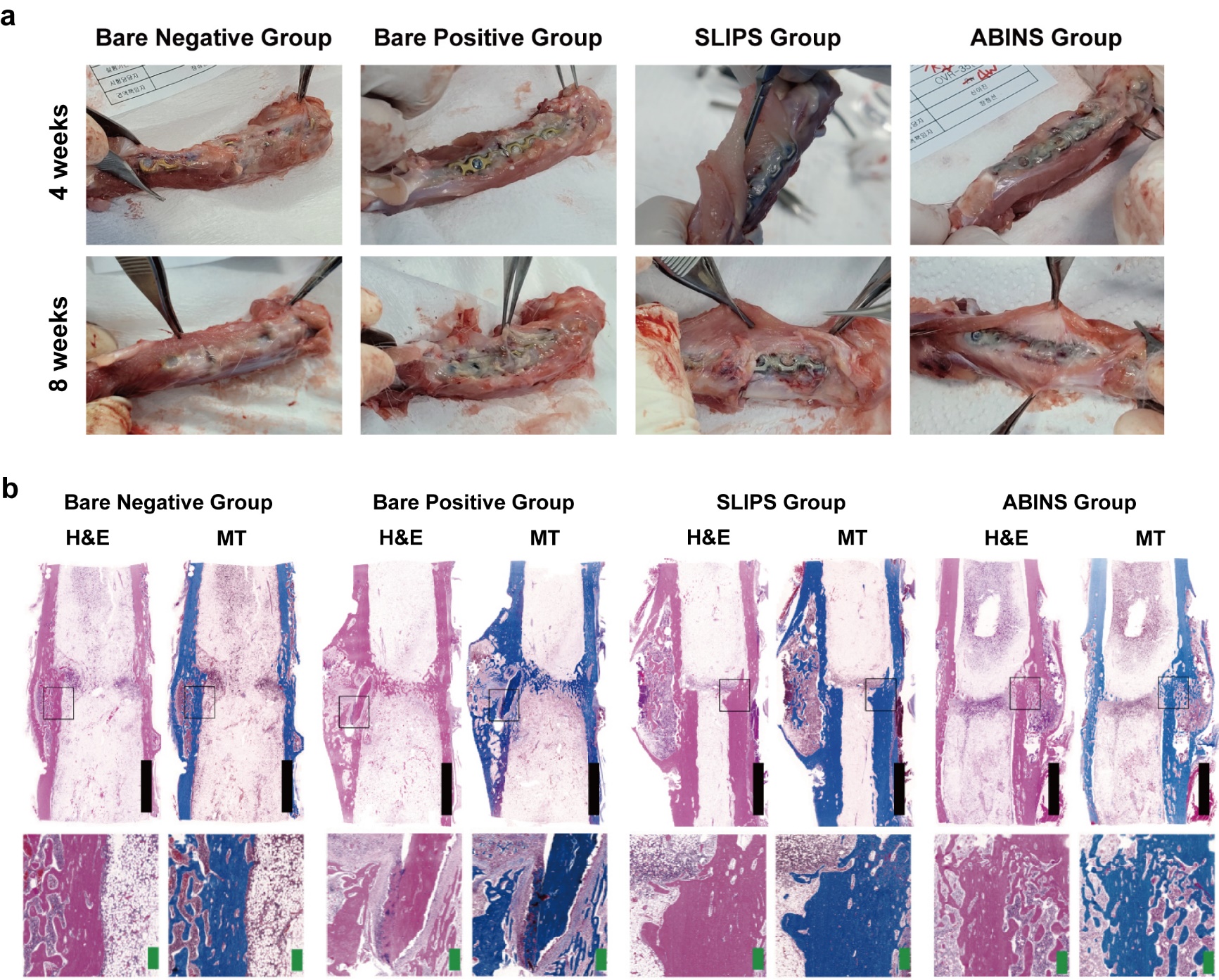


**Supplementary Figure 6.** H&E and MT staining. Sliced tissue samples (8 weeks sacrifice group) were stained with H&E and MT staining solutions. In the bare negative, SLIPS, and ABINS groups, the immunological reactions were sparse. However, in the bare positive group, some inflammations of the fractured bones were observed. (Upper images= overall stained tissue images, lower images= magnified images) Scale bars in black = 5 mm , scale bars in green= 0.5 mm)


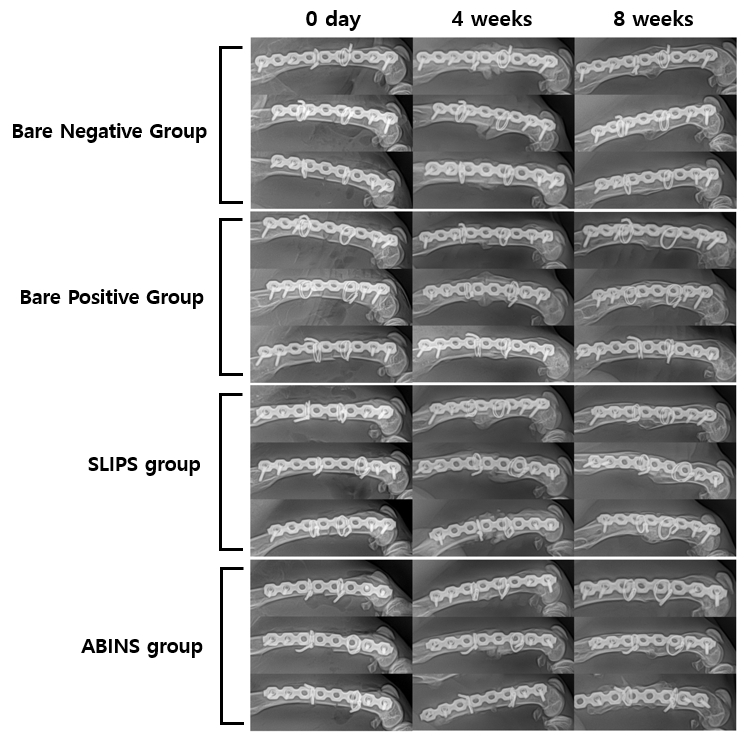
Supplementary figure 7

**Supplementary Figure 7.** Results of osteosynthesis during study period. At 4 weeks following fracture surgery, all four groups showed signs of callus formation, which means initiating secondary bone healing process. At 8 weeks, all four groups showed successful bone healing with cortical bridge formations. Each group includes 3 individual rabbits.

Supplementary Figure 8


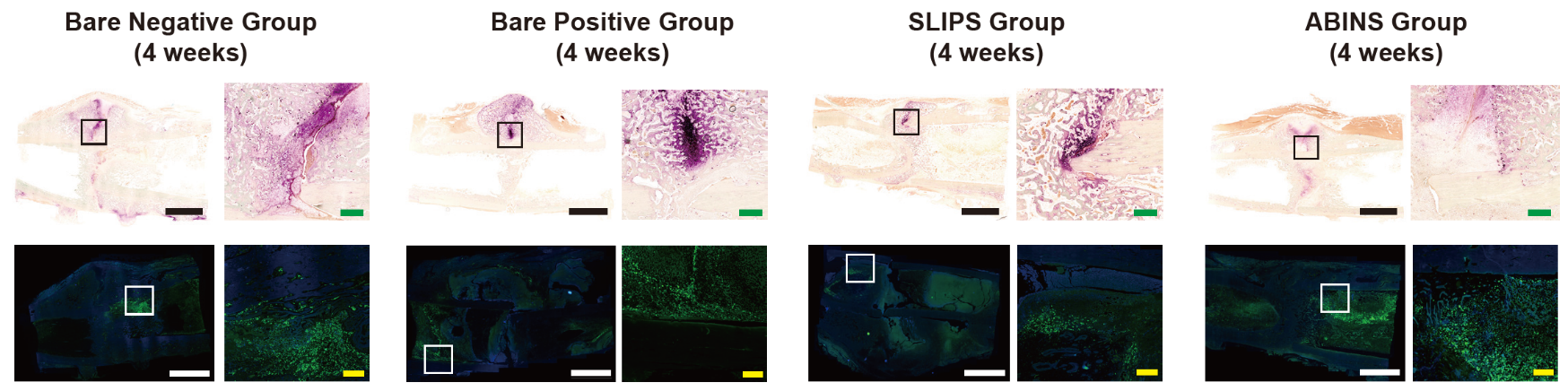


**Supplementary Figure 8.** Osteoclast-specific and osteoblast-specific staining on tissue slides to visualize the bone healing process. Osteoclast activities and osteoblast activities for each group were assessed with osteoclast-specific tartrate-resistant acid phosphatase (TRAP) staining (upper images) and osteoblast-specific alkaline phosphatase (ALP) immunofluorescence staining (lower images). The magnified images of the right side for each group were boxed in the overview images on the left. Scale bars in black or in white= 5mm, scale bars in green or in yellow= 0.5 mm.

Supplementary figure 9

**
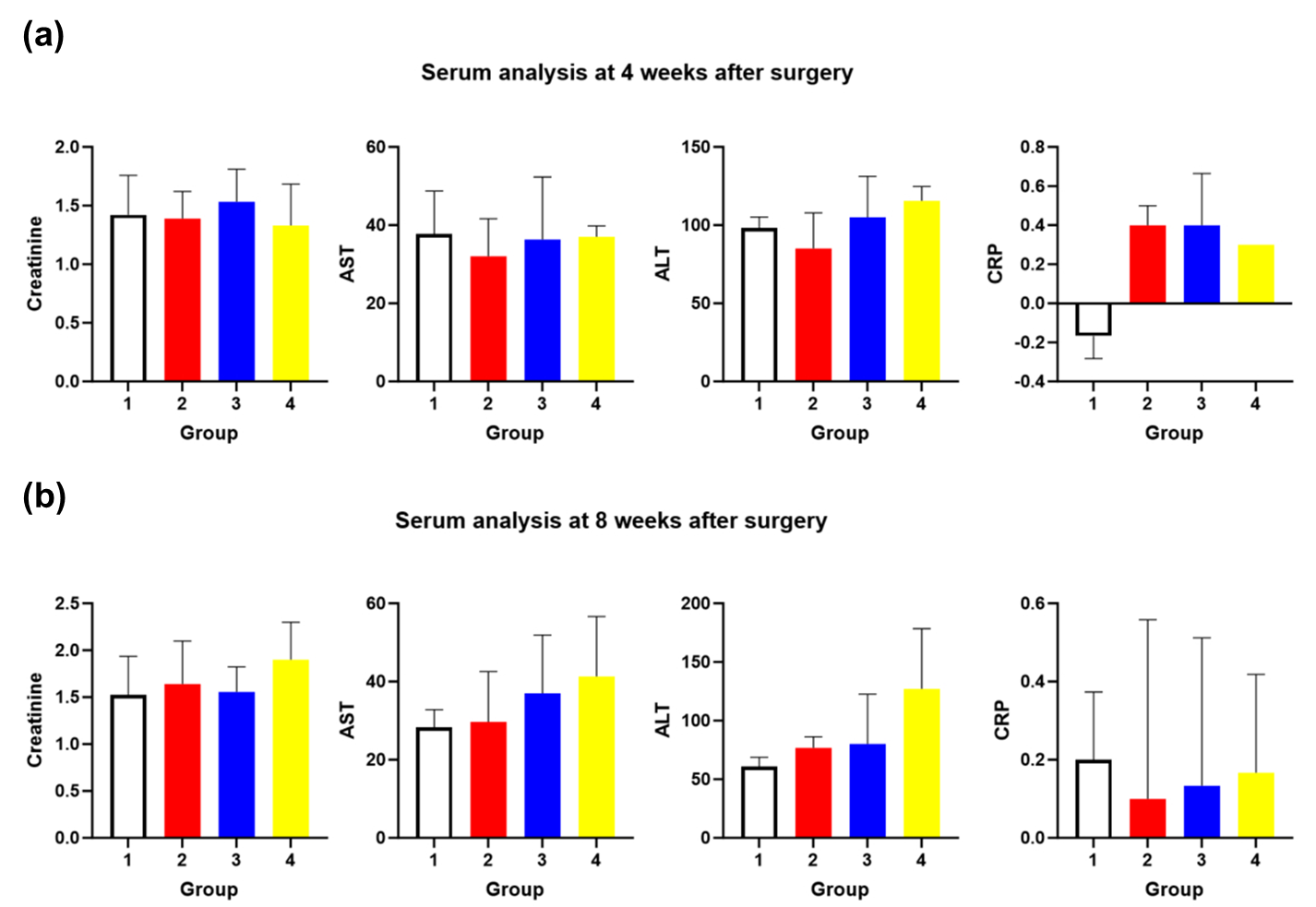
**

**Supplementary Figure 9.** Results of blood serum analysis. (a) At four weeks, no differences were found in serum creatinine, AST, ALT level. Bare negative group showed lowest serum CRP level although not statistically significant. (b) At eight weeks, all values showed no significant differences; Group 1= Bare negative, Group 2= Bare positive, Group 3= SLIPS, Group 4= ABINS.

**Supplementary Methods**

**In vivo surgical protocols**

The animals were anesthetized with intravenous injection of zoletile (Virbac, France) and Rompun (Bayer, Germany) for each 1ml through ear vein for induction of anesthesia. Then, 2% isoflurane with 70% of medical oxygen was delivered through an animal mask system to maintain anesthesia. The femur bone was approached through the lateral aspect. All the skin hairs on the lateral side of the thigh were removed with a motorized shaver and sterilized with povidone-iodine.

After palpation of the femur bone location, about 5 to 7cm-long incision was made directly on the femur bone on the lateral side of the thigh of the animal. After incising muscle fascia, the whole femur bone can be easily exposed through the plane between two adjacent muscles on the thigh. Bleedings were carefully coagulated especially around the distal femur head area where moderate-sized vessels were running. Then, appropriate lengths of the surgical plates were determined on the femur bone, then cut with a plate-cutting device provided by the implant company. A complete fracture was made about one-third portion proximal to the femur bone with a motorized saw (Supplementary Figure 9a). Temporal intramedullary wire was inserted through the fracture site and penetrated through the knee joint (Supplementary Figure 9b). Then, after fracture reduction, the metal wire was retrogradely reinserted into the most proximal portion of the femur bone for temporal fixation of fracture (Supplementary Figure 9c). Then, pre-cut surgical plates were placed on the femur, and most proximal and distal screws were inserted first (Supplementary Figure 9d). The surgical plates have been prepared with biofilms of *P. aeruginosa* (NCCP 15783). Screw holes were made with a motor drill, and screws were inserted after measuring the depth of the screw hole. Then, the 3^rd^ and 4th screws were inserted with one to two holes distance jumping to the first and second screws (Supplementary Figure 9e). Uni-cortical screws were inserted to reduce the risk of the periprosthetic fracture occurring through screw holes made on the bone. Afterward, metal tension wires were wrapped proximal and distal to the fracture site (Supplementary Figure 9f). Finally, temporally inserted intramedullary metal wire was removed. Before suture closure of the surgical wound, additional 2ul of bacteria (*P. aeruginosa* suspension) were inoculated on the surgical site (10^6 CFU/ml) (Supplementary Figure 9g). This is for simulating surgical site infection that could happen during surgery during practical clinical scenario. Then, the muscle fascia was repaired with an absorbable suture, and the skin was closed with a non-absorbable suture (Supplementary Figure 9h).

**
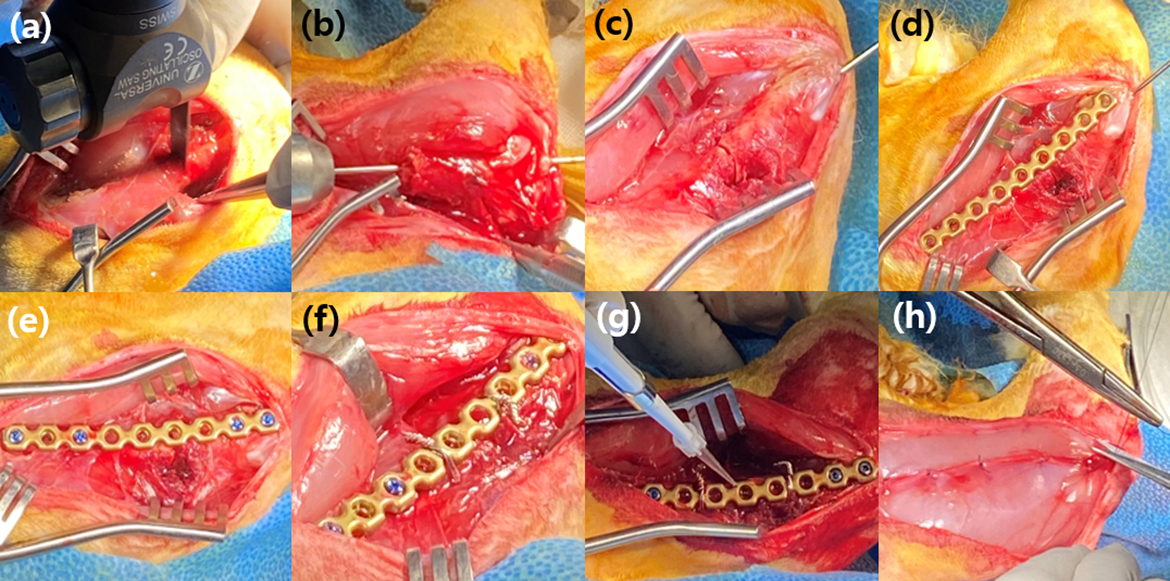
**

**Supplementary Method Figure.** Procedures for rabbit femur infected fracture surgical fixation model

**In vitro cytotoxicity and cell adhesion tests of the surface coating**

For cytotoxicity and cell adhesion tests, mouse fibroblast and macrophage cells were used. Mouse fibroblast (3T3-L1) and macrophage (Raw264.7) cell line purchased from Korean cell line bank (KCLB) were cultured and maintained in the 10% FBS-containing DMEM. Cell adhesion was determined by direct seeding the cells at a density of 1x10^5^ cells/cm^3^ onto coated surface with the indicated condition. After 24-hour incubation, culture supernatant was removed, and the cell-adhering surface was washed twice with PBS, followed by CCK-8 assay (Dojindo). To evaluate cytotoxicity, cell culture trans-well inserts (Falcon) were placed in each well of cell culture well plate, where coated surfaces were present at the bottom. Then, cell suspension was added to the inserts at a density of 1x10^5^ cells/cm^3^. After 48-hour incubation, the inserts were transferred to each well of new well plates and washed twice with PBS, followed by CCK-8 assay. CCK-8 assay was performed as manufacturer’s protocol. Briefly, CCK-8 solution was added to the cell culture media at 1/10 ratio, and incubated at 37ºC for 1 hour, followed by reading absorbance at 450 nm using spectrophotometer. Experiments were repeated twice and each experiment includes triplicates. The absorbance was converted to percentage by dividing optical density (OD) value of each group divided that of control group.

For the imaging with confocal microscopy, cell culture was performed using the same method as previously described above for the cytotoxicity assay. For immunofluorescence, cells were fixed with cold methanol for 5 minutes at -20°C, permeabilized with 0.1% Triton X-100 for 10 minutes, and blocked with 2% normal goat serum (NGS) for 1 hour at room temperature. The cells were then incubated with the beta-actin antibody (Millipore, MAB1501R) at a dilution of 1:500 overnight at 4°C. Following washes with phosphate-buffered saline (PBS), cells were incubated with the FSD™ 488-conjugated secondary antibody (BioActs, RSA1145) at a dilution of 1:500 for 1 hour at room temperature. Immunofluorescence images were acquired using a confocal microscope (LSM900, Carl Zeiss) with a ×20 objective lens and processed using the Zeiss ZEN lite software.

**Serum enzyme level analysis during the recovery period**

Blood analyses were done at two time points (4 and 8 weeks before sacrificing the animal). To analyze the kidney and liver function, serum creatinine, aspartate transaminase (AST), alanine aminotransferase (ALT) levels were investigated, and to check the systemic inflammation level, serum C-reactive protein (CRP) levels were also examined at four, eight weeks following surgery in the blood. Ten ml of venous blood were taken from inferior vena cava just before sacrificing the subjects.

**Bacterial infection and inflammation analyses with histology staining**

For immunofluorescence staining for alkaline phosphatase (ALP), a biomarker of osteoblast, 5-μm tissue sections were first incubated in 10 mM sodium citrate buffer (c9999-100ML, Sigma Aldrich) at 99°C for 20 minutes for antigen retrieval. Blocking was performed in 3 % BSA in TBST for 1h. and then incubated with ALP primary antibodies (Santa Cruz Biotechnology, sc-271431) overnight. Followed by three washes with TBST and incubation for 1 hour with fluoresce probe–labeled secondary antibody (m-IgG Fc BP-FITC: sc-533651, Santa Cruz Biotechnology). Sections were counterstained with Ultra Cruz Mounting Medium with DAPI (Hard-set Mounting Medium with DAPI-Ultra, sc-359850, Santacruz Biotechnology) and covered by a coverslip. Fluorescent images were obtained with the slide scanner as described above.

For TRAP staining, the slides were dipped into a pre-warmed TRAP staining solution mix (of 200 mL of TRAP Basic incubation medium, 120mg of Fast red violet LB salt (Sigma, F-3381), and 1mL of naphthol AS-MX phosphate substrate mix) and incubated at 37 °C for 24 h. After 24 h, they were rinsed in distilled water. In the next step, they were counterstained with 0.02% Fast Green (CAS# 2353-45-9) for 30 sec and rinsed quickly in distilled water. After that, they were quickly dehydrated into a series of alcohol of 70%, 80%, 90%, and 100%. Clear in Xylene, and the slides were mounted with coverslips by using the mounting solution (Epredia™, #6769007). Finally, the slide images were analyzed through the slide scanner as above.
